# An overview of glioblastoma multiforme and temozolomide resistance: can LC-MS-based proteomics reveal the fundamental mechanism of temozolomide resistance?

**DOI:** 10.3389/fonc.2023.1166207

**Published:** 2023-04-26

**Authors:** Milan Teraiya, Helene Perreault, Vincent C. Chen

**Affiliations:** ^1^ Chemistry Department, University of Manitoba, Winnipeg, MB, Canada; ^2^ Chemistry Department, Brandon University, Brandon, MB, Canada

**Keywords:** cellular proteomics, glioblastoma multiforme (GBM), temozolomide resistance (TMZ resistance), IDH, chemoresistance, MGMT, LC-MS

## Abstract

Glioblastoma multiforme (GBM) is a primary type of lethal brain tumor. Over the last two decades, temozolomide (TMZ) has remained the primary chemotherapy for GBM. However, TMZ resistance in GBM constitutes an underlying factor contributing to high rates of mortality. Despite intense efforts to understand the mechanisms of therapeutic resistance, there is currently a poor understanding of the molecular processes of drug resistance. For TMZ, several mechanisms linked to therapeutic resistance have been proposed. In the past decade, significant progress in the field of mass spectrometry-based proteomics has been made. This review article discusses the molecular drivers of GBM, within the context of TMZ resistance with a particular emphasis on the potential benefits and insights of using global proteomic techniques.

## 1 Introduction

Malignant tumors of the central nervous system (CNS) are difficult to treat and often result in poor overall patient survival (1). Glioblastoma multiforme (GBM) is a particularly aggressive tumor of the CNS. Its overall median survival of GBM patients is 12–18 months (4.6% survival rate at 5 years) (2). GBM cells are highly proliferative and infiltrative, which limits the possibility of complete tumor removal by surgical resection. In terms of clinical presentation, GBM patients experience persistent weakness, loss of vision, and alteration of speech (3). Around 14% of brain and CNS tumors belong to the category of GBM (4). Given the rare metastatic behavior of CNS tumors, the severity of the disease is graded from 1 to 4 by the World Health Organization classification of CNS tumors (5). Grade 4 glioma, also known as *glioblastoma multiforme (GBM)*, is the most malignant type of primary CNS tumor (1).

The blood–brain barrier (BBB) involves astrocytes, neurons, and endothelial cells which regulate the transport of molecules into the brain. The BBB imposes the selective permeability of molecules. It is thought that lipophilic molecules with a molecular weight (MW) of less than 400 Da might have the possibility to pass through the BBB, whereas large and hydrophilic molecules are generally restricted (6). Hence, the BBB is an obstacle for transporting chemotherapeutics targeting various types of brain cancers including GBM (7). As a BBB-permeable drug, temozolomide (TMZ), commercially known as Temodar™, is the principal drug used to treat GBM, alongside radiation and surgical resection. Orally administered, TMZ is a DNA alkylating agent which breaks the DNA chains by attaching a methyl group to guanine at the oxygen-6 (O6), nitrogen-7 (N7), and adenine-3 (N3) positions. This generates the cytotoxic bases O6-methylguanine (O6-MG), N7-methylguanine (N7-MG), and N3-methyladenine (N3-MA) that manifest in a beneficial, clinical effect (8). During DNA replication, the mismatched base pairs induce cell-cycle arrest in the G2/M phase to induce death (2). In this regard, malignant tumors adapt. Here, a population of tumors may already possess “intrinsic” or “innate” resistance. Alternatively, tumor cells can also “acquire” or modify cellular networks to bypass the actions of the therapeutic agent. In both cases, continued exposure to these conditions applies selective pressures for cells to self-select and evolve into drug-resistant tumors (9, 10). Indeed, studies of TMZ-resistant GBMs demonstrate the activation of pathways responsible for DNA repair including 1) O6-methylguanine-DNA-methyltransferase (MGMT), 2) DNA mismatched repair (MMR) system, and 3) base excision repair (BER, the poly(ADP)-ribose polymerase (PARP) pathway) (11). Understanding of these networks and mechanisms linked to these processes may provide avenues for the development of new drugs and adjuvants.

Proteins are significant for their roles in pathophysiology, cellular biology, and molecular functions. The detailed study of the proteome is called proteomics, which investigates protein abundance, protein–protein interactions (PPI), cellular localization and functions, posttranslational modifications, and cellular signaling. Proteomics studies may be conducted under normal physiological conditions or under stress or pathological conditions (12). Conventional proteomics-based techniques such as two-dimensional polyacrylamide gel electrophoresis (2D-PAGE) and 2D differential in-gel electrophoresis (2D-DIGE) have been complemented with state-of-the-art high-throughput techniques such as liquid chromatography combined with high-resolution mass spectrometry (LC-MS). These improvements have been very useful in differentiating proteins and their expression levels in different biological systems (13). Protein identification and quantitation using these techniques can reveal differential expression of proteins and biomarkers (14).

Mass spectrometry-based proteomics can provide important insight into TMZ resistance phenotypes. The involvement of proteins in molecular function, signaling cascade, and protein–protein interactions can reveal crucial information (15). As GBM resistance to TMZ involves complex mechanisms, analyzing a large number of proteomic samples from different laboratories would provide a good chance to identify biomarkers with confidence to help in further efforts to understand TMZ resistance (16). The creation of an international LC-MS proteomics database of TMZ-resistant cells would be an important step in this direction, and this topic will be discussed at the end of this article.

## 2 Background

### 2.1 Brief details on *glioblastoma multiforme* and tumors of the CNS

As per the International Agency for Research on Cancer (IARC) database, brain and CNS cancers contribute to 3% of deaths among all other types of cancers (17). According to IARC, by 2040 there will be almost a double-fold increase (~46.5%) in deaths due to CNS tumors. In a more optimistic outlook, advances in immunohistology and molecular biology have revolutionized the understanding of CNS tumors, which is crucial for the development of therapies. The first edition of histological typing of CNS tumors was presented in 1979 to the WHO in Geneva (18). In 1993, the second edition revealed that histological typing of the tumors based on immunohistology was very useful (19). In 2000, a WHO working group classified CNS tumors based on histology and considered additional genetic background information in order to clarify disease diagnosis. The 2000 edition included data based on science, clinical signs and symptoms, imaging, and survival predictions (20). Further developments in molecular biology, mainly in genetics, provided characteristic molecular information on CNS tumors. Hence, in 2016 the fourth edition was released with considerable information on the latest pieces of evidence, combining histology with genetics, to understand various types of CNS tumors. A new classification of CNS tumors was published by WHO in 2016 (1). This revised classification incorporated molecular features and histogenesis of CNS tumors, whereas the previous WHO classification (2007) was based only on histological characteristics. The 2016 classification represents a paradigm shift in neuro-oncology and provides well-defined criteria for tumor definition, characterization, nomenclature, diagnosis, and treatment options. These drastic changes constituted major steps forward for strategic planning of patient treatments.

**Figure 1:**
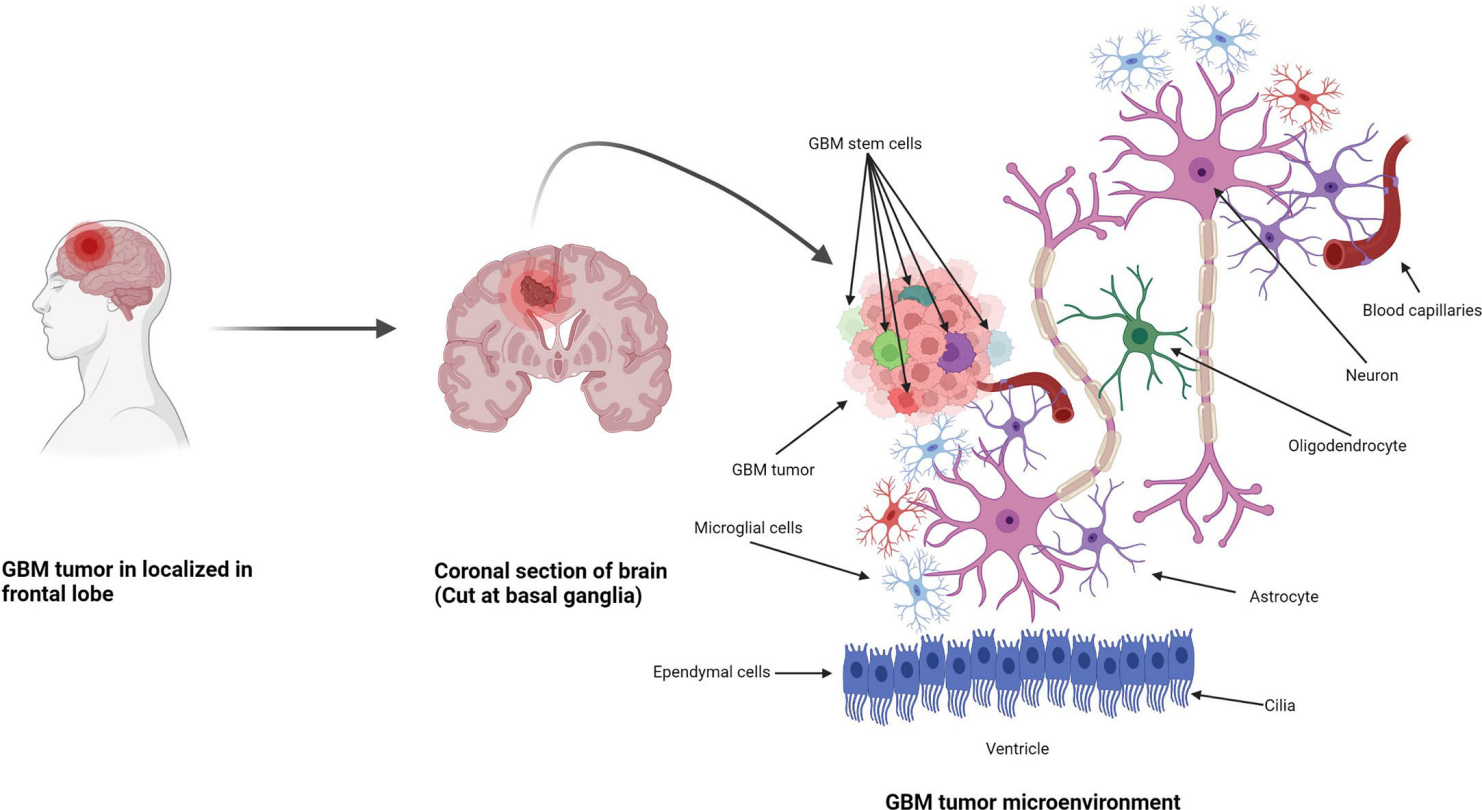
Formation of GBM tumors and their microenvironment. GBM tumors are often diagnosed in the frontal lobe, temporal lobe, and cerebellum (21). GBM tumor cells are composed of heterogenous cell populations (containing GMB stem cell markers (GSCs) in green, dark red, green dark green, purple, etc.) with properties of neural stem-like cells (NSCs): self-renewal, generation into differentiated GBM cells (22). The GBM microenvironment contains a variety of glial-type-associated cells including microglia and astrocytes, which are highly associated with glioma pathophysiology (23, 24).

Anatomically, the CNS is made up of two types of cells: neurons and neuroglia. Neuroglia are non-neuronal cells representing half of the volume of the brain. Their function is to support, nourish, and protect the neurons, and they have the ability to divide and grow during their lifetime (**Figure 1**) (25). Neuroglia are also known as glia and are the most abundant cells in the CNS. There are four types of neuroglial cells: astrocytes, oligodendrocytes, microglia, and ependymal cells. Tumors arising from these cells are known as gliomas and are highly malignant in nature. A proportion of 29.1% of primary brain and other CNS tumors are malignant, and 70.9% are non-malignant. In the USA only, there was a projection of 88,970 new diagnoses of CNS tumors in 2022 (26). Also in the USA, an average of 16,606 deaths per year are reported, corresponding to 4.43 per 100,000 people (26). Glioblastoma multiforme (GBM) was the highest type of malignant tumor reported (14.3% of all tumors) with more frequency in men than in women. After diagnosis, the 5-year survival rate for patients with malignant brain and CNS tumors was 66.9% (26). Among all CNS tumors, GBM had the lowest median observed survival rate of only 8 months (26).

**Figure 2:**
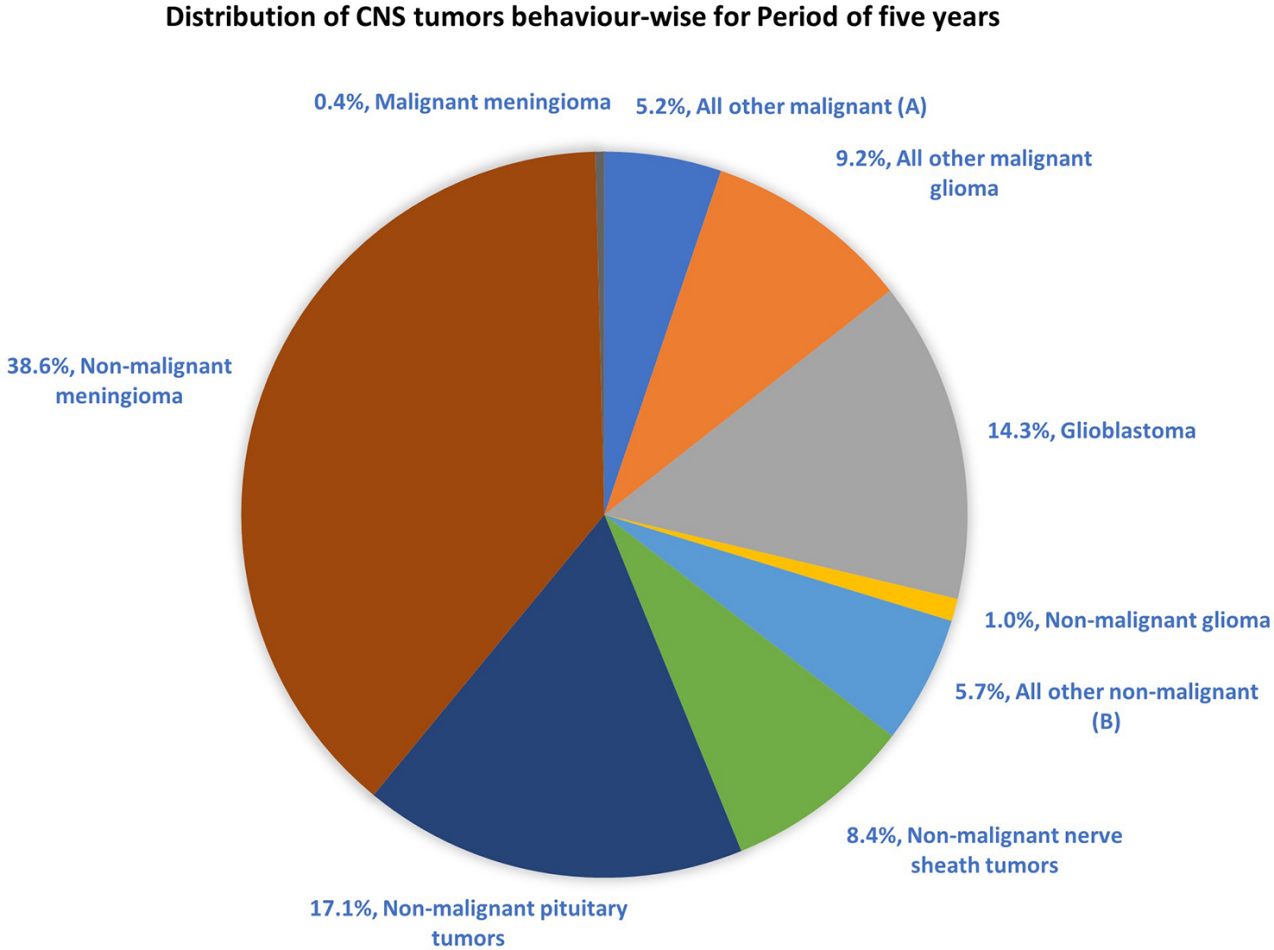
Distribution of primary brain and other CNS tumors by behavior (5-year total = 431,773 cases), CBTRUS statistical report (2014–2018): Malignant tumors are 29.1% and non-malignant 70.9% (26). Remark: The total percentage will not be 100, as rounding off was performed; **(A, B)** contain histologically different behaviors.

The most common malignant tumors are due to GBM, representing 14.3% of all tumors arising in brain and CNS. In contrast, non-malignant tumors count for 38.3% of all diagnosed tumors (26). Gender-wise, GBM is more common in males, whereas meningioma is more common in females. The 5-year relative survival rate for malignant brain and CNS tumor was 35.6% versus 91.8% for non-malignant (26). **Figure 2** shows the distribution of types of tumors and highlights the difference between survival rates of patients afflicted with non-malignant and malignant CNS/brain tumors (26).

### 2.2 WHO CNS classification (2016, 2021) and isocitrate dehydrogenase profile in GBM: A major reform

In 2016, a WHO workgroup including neurologists, oncologists, histopathologists, and geneticists investigated brain and CNS tumors with the clear aim of classifying tumors based on the most advanced knowledge and on concerns expressed within workgroup members. Major key changes were incorporated into the first edition of classification based on histology in 1979, immunohistochemistry in 1993, genetics in 2000, and histopathology and genetics combined in 2007 (18, 19, 27). GBM tumors are well characterized clinically, and the 2016 CNS WHO classification is an example of how understanding the molecular biology of tumors helps to advance diagnostic and treatment trajectories (28). For GBM, incorporation of novel criteria for classification such as IHD1/IDH2 wild-type and IHD1/IDH2 (where IDH is isocitrate dehydrogenase) mutant genes led to newer grading for CNS neoplasm in GBM with improved diagnosis and focused treatment, providing superior prognosis with regard to clinical outcomes and patient survival rates (29).

## 3 Mechanism of action of temozolomide in GBM

**Figure 3:**
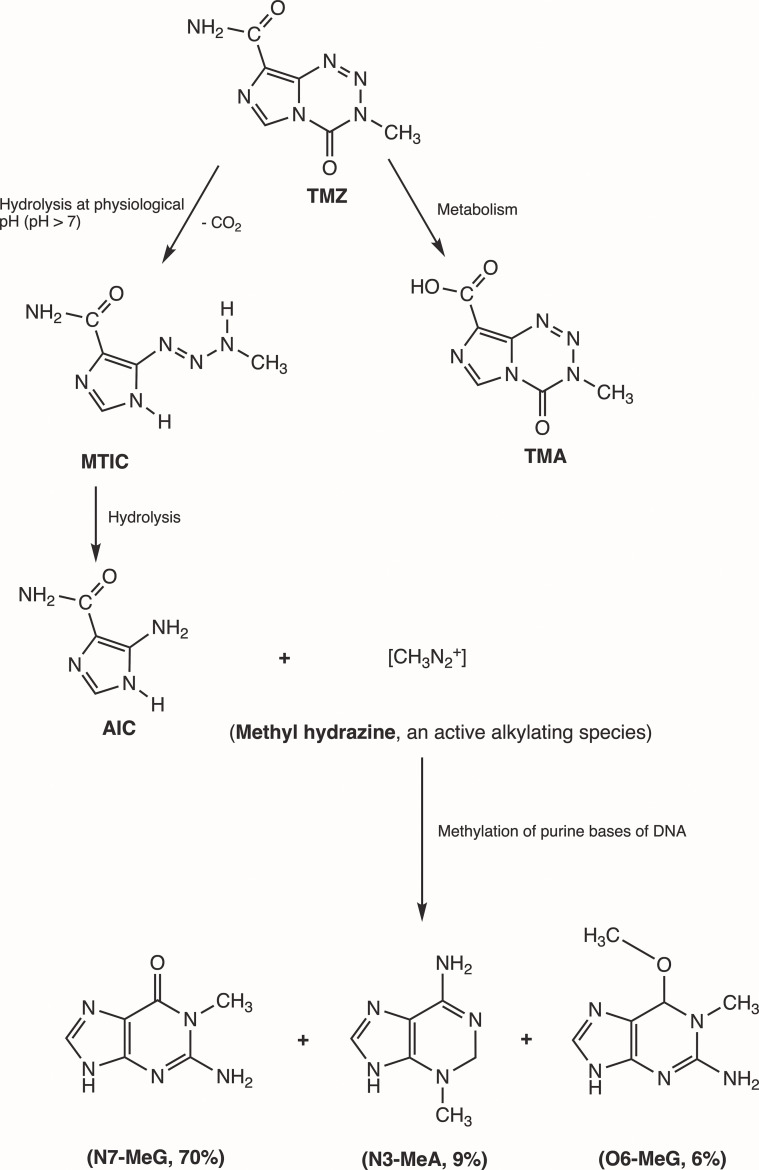
Mechanism of TMZ after oral administration. Activation of prodrug (TMZ) occurs at physiological pH (>7) after oral intake. TMZ is stable in an acidic environment (pH<7) and degrades rapidly by hydrolysis at basic pH. TMZ hydrolyzation results in MTIC *via* decarboxylation and further hydrolysis produces AIC and methyl hydrazine (DNA methylating species). The pH of brain tumors is alkaline compared with healthy surrounding tissues, resulting in increased activation of TMZ within the tumor environment (35). (TMZ, temozolomide (3-methyl-4-oxo-3H,4H-imidazo[4,3-d][1,2,3,5]tetrazine-8-carboxamide); TMA, temozolomide acid; MTIC, 5-(3-methyltriazen-1-yl)-imidazole-4-carboxamide; AIC, 5-aminoimidazole-4-carboxamide; N7-MeG, methylation at N7 position of guanine; N3-MeA, methylation at N3 position of adanine; O6-MeG; methylation at O6 position of guanine).

Initially, Steven et al. synthesized the first-generation antineoplastic agent mitozolomide (8-carbamoyl-3-(2-chloroethyl)imidazo [5,1-d]-1,2,3,5-tetrazin-4(3H)-one. The effect of this agent was studied in L1210 mouse leukemia cells (30). Mitozolomide exerts DNA cross-linking through ethylene bridge formation. In the 1990s, the new second-generation imidazotetrazine-based chemotherapeutic prodrug, TMZ, emerged. The chemical designation of TMZ is 3-methyl-4-oxoimidaz[5,1-d][1,2,3,5]tetrazine-8-carboxamide. TMZ is part of a new class of alkylating agents with an imidazole ring (31). As a prodrug, TMZ itself is not active and does not require hepatic metabolism to create the active metabolite methyltriazen-1-yl imidazole-4-carboxamide (MTIC). The drug TMZ is absorbed efficiently after oral administration. It has time dependent antitumor activity and crosses the BBB. TMZ gets hydrolyzed at physiological pH (pH >7) into MTIC, which degrades and generates the reactive DNA methylating species methyl hydrazine (30, 32, 33). TMZ shows cytotoxicity only once it modifies its targets by addition of methyl groups at N7 (>70%) and O6 (6%) sites of guanine and N3 (9%) sites of adenine in genomic DNA (34) (**Figure 3**). Although occurring in a low proportion (7%), methylation of guanine at O6 (O6-MeG) is cytotoxic, mutagenic, and critical for TMZ-induced cytotoxicity (35). This methylation step damages DNA, and GBM cells will use different pathways to treat these modifications from TMZ.

TMZ modification of GBM cells at the O6 site of guanine leads to DNA replication errors due to mismatched bases. Instantly, the mismatch repair (MMR) system will interrupt the replication process. When MMR enzymes recognize the mispaired thymine on the daughter strand, they will excise the mispaired fragment, whereas O6-MeG remains in the template strand. After the MMR’s attempt to remove the O6-MeG adduct, single- and double-stranded breaks in the DNA are produced, triggering cell death by apoptosis (31). Methylation of the N7 site on guanine accounts for 70% of TMZ modification in cells, but no corresponding cytotoxicity has been found. In 9% of TMZ treatment cases, N3-methyladenine is extremely toxic for cells and can block the progression of DNA replication or cause chromosome aberration, thus killing GBM cells (36). Cytotoxicity of TMZ depends on the normal MMR mechanism, and GBM cells are sensitized by TMZ when the DNA-MMR mechanism is triggered, causing double-strand DNA to break and leading to programmed cell death (37).

## 4 Temozolomide resistance in GBM

**Figure 4:**
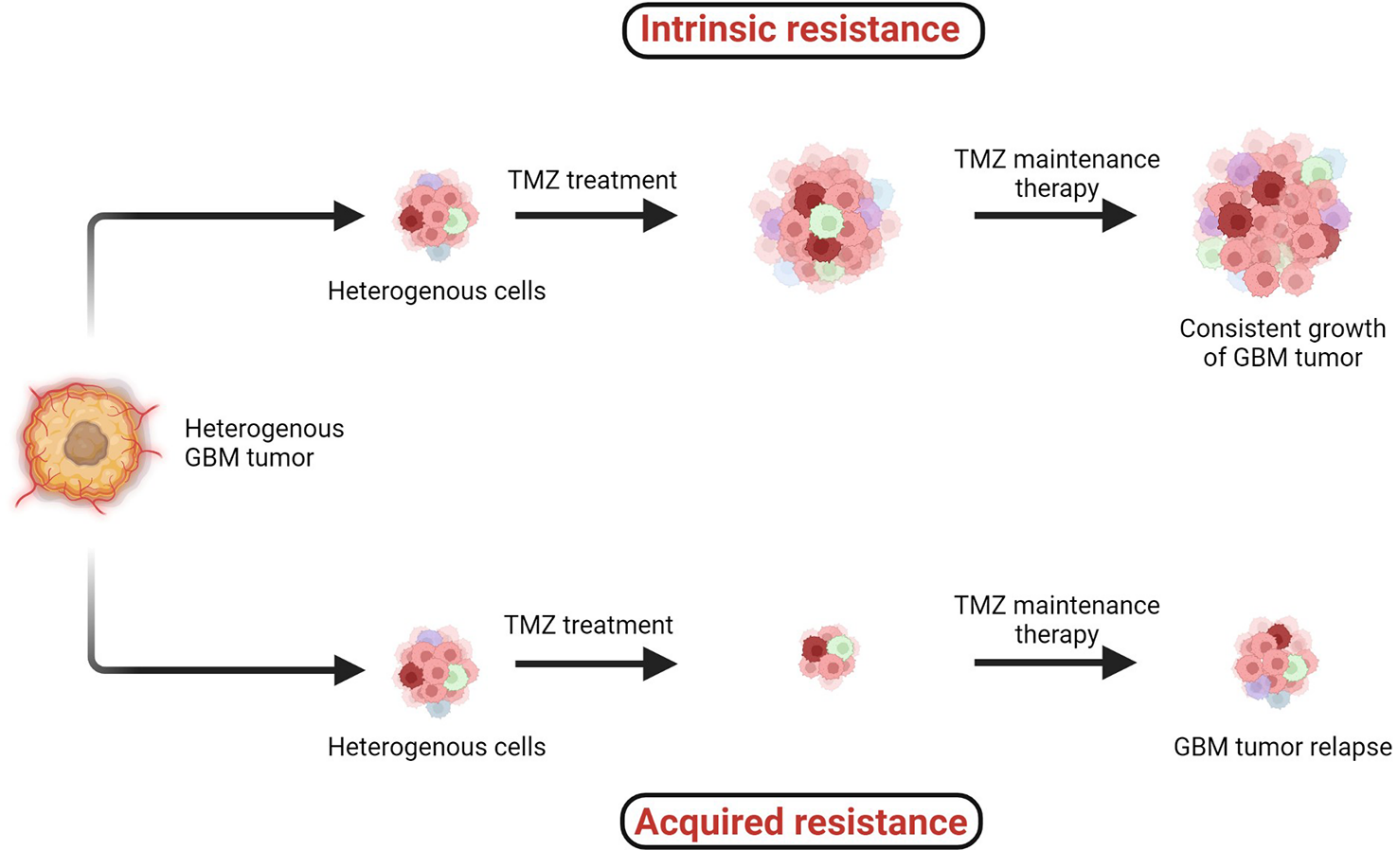
Two major types of resistance development: (1) intrinsic and (2) acquired resistance. Intrinsic resistant tumors contain very small amounts of or no antitumor T cells, creating natural resistance against therapies. Acquired resistance is resistance development against the therapy after clinical treatments are given (38–41).

TMZ chemotherapy administered with radiotherapy offers significantly superior prognosis for GBM patients, and hence it is widely considered as a first-line chemotherapy regimen to treat GBM. However, two major types of resistance against the chemo- and radiotherapies, (1) intrinsic and (2) acquired, remain persistent challenges across all cancers including GBM (**Figure 4**) (38, 41).

When the specific tumorigenic subpopulation of GBM cells is intrinsically resistant, these cells maintain uncontrolled cellular proliferation before, during, and after therapeutic treatment and hence the tumor mass keeps growing. In the case of acquired resistance, a rare population of cells acquires resistance against the therapies (including radio- and chemo-) *via* genetic mutations or abnormal alterations in cell signaling pathways. Generally in the acquired resistance phenotype, clinical benefits of treatment are observed initially and the tumor mass is reduced; however, after a certain period of treatment due to acquisition of resistance, the tumor relapses (39).

Several cell lines have been studied extensively to understand the molecular mechanisms causing TMZ resistance in GBM. This phenomenon remains insufficiently understood as multiple molecular mechanisms are involved and need to be investigated (42). Several theories of TMZ resistance have been published; however, gaps in further detailed understanding still remain (2, 43, 44).

TMZ has been used as a frontline DNA methylating agent for GBM since its approval by USFDA in 2005. In terms of cytotoxicity, no other drugs have been found as efficient as TMZ. However, GBM cells involving high extents of DNA repair can reverse the methylation efficiency of TMZ, in which case GBM cells can become resistant against TMZ therapy. A challenge still persists, as for 50% of patients who receive TMZ as primary chemotherapy, poor survival rates are observed due to TMZ resistance in GBM (42).

Three major types of DNA repair mechanisms are responsible for TMZ resistance: MGMT, MMR, and base excision repair (BER, the poly (ADP)-ribose polymerase (PARP) pathway) (11). The primary resistance mechanism against TMZ is directly linked with high MGMT expression, whereas a secondary mechanism is linked to the MMR system in cells lacking MGMT (45–47).^,,^ The third mechanism is the PARP pathway, which mainly involves the removal of N7-methylguanine and N3-methyladenine adducts (42). This BER pathway has minimal effects compared with MGMT and MMR. This is because the removal of N7- and N3-methyl adducts does not cause DNA double-strand breaks. When MGMT, alkylpurine-DNA-glycosylase (APNG), and BER protein are expressed, GBM cells are resistant to TMZ (42).

### 4.1 O^6^-Methylguanine-DNA methyltransferase

In GBM, MGMT is a significant role player in developing resistance against chemotherapies, including TMZ. MGMT plays a suicidal role in repairing methylation of O6-MeG lesions, resulting in the decreased cytotoxic effect of TMZ. MGMT reverses the mechanism of TMZ by removing the methyl group O6-MeG and restores DNA into its original state (48). TMZ induces DNA methylation by generating O6-methylguanine, which triggers cytotoxicity and apoptosis (30, 32). The MGMT gene is located on chromosome 10q26 and encodes a DNA-repair protein, which eliminates methyl groups from the O6 position of guanine, thus avoiding gene mutation, cell death, and tumorigenesis caused by alkylating agents (46). The epigenetic regulation of specific sites of MGMT CpG islands influences MGMT transcription (49). CpG islands are composed of short stretches of DNA with a high cytosine 5′ to guanine content, separated by a phosphodiester bond. Chemotherapy and radiotherapy may modulate the methylation level of the MGMT gene as well as protein expression. Overexpression of MGMT is an important mechanism of TMZ resistance.

**Figure 5:**
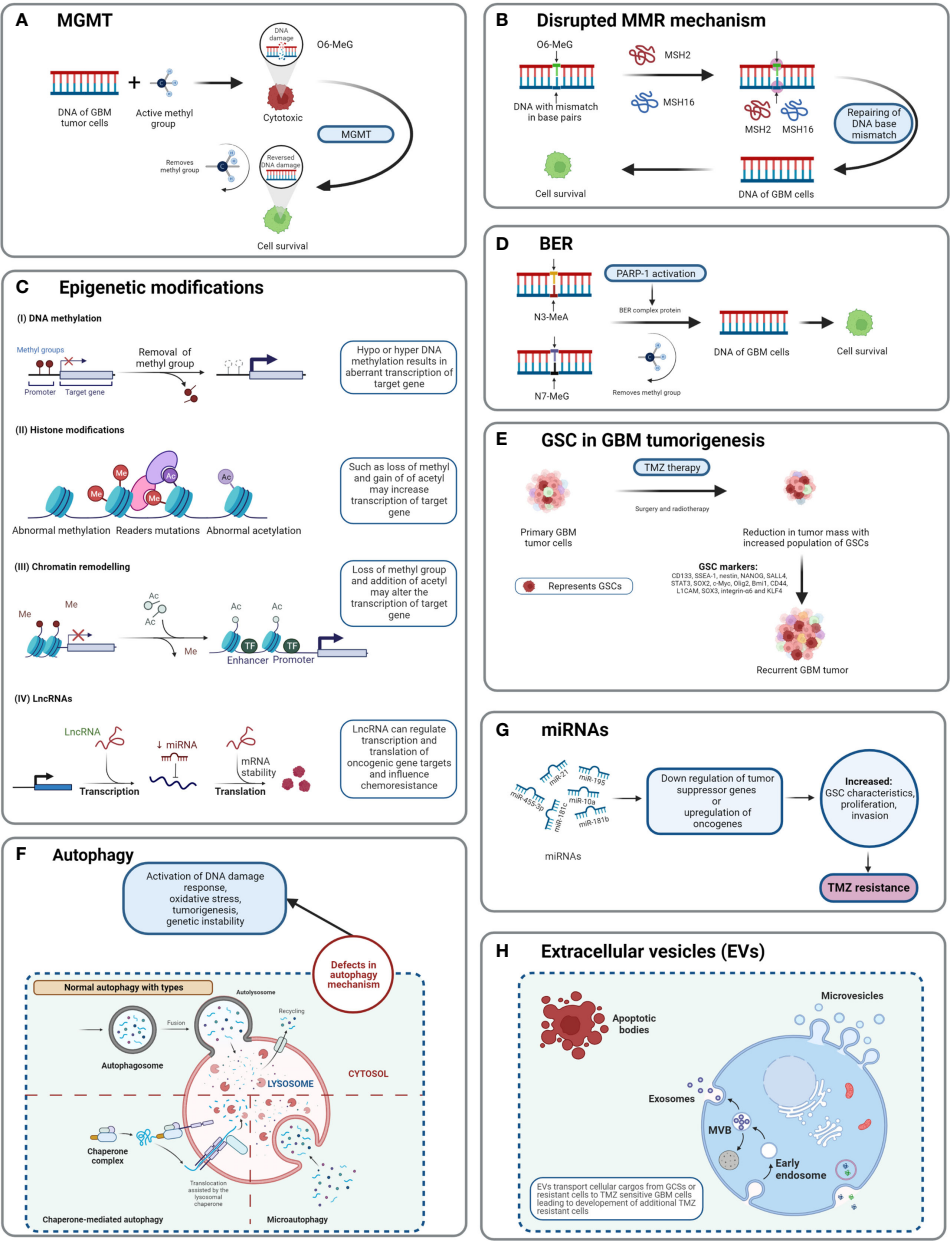
Detailed TMZ resistance mechanism in GBM. **(A)** Enzyme MGMT reverses the methylation O6-MeG by removing a methyl group, leading to increased cellular survival of GBM cells (50). **(B)** Disruption in the MMR mechanism repairs the mismatch between DNA bases (51). **(C)** Major epigenetic alterations include DNA methylation, histone modifications, chromatin remodeling, and lncRNAs (52). **(D)** The BER system removes or repairs DNA nucleotides. N3-MeA and N7-MeG are repaired by the BER mechanism (2). **(E)** GCSs are diverse and lead to generation of TMZ-resistant cells with self-renewal capabilities (52). **(F)** Defects in normal autophagy contribute to resistance development against TMZ (52). **(G)** The expression levels of miRNAs alter gene regulation, causing TMZ resistance (52). **(H)** Extracellular vesicles (EVs) are capable of regulating TMZ resistance related to biological material and cellular cargo (52).

MGMT can reverse the methylation of TMZ on the O6-MeG position by transferring the methyl group to an internal cysteine residue, which leads to no cytotoxicity from TMZ, thus preventing cells from destruction (TMZ resistance) (**Figure 5A**) (53) (54)., Monica et al. and Rodrigo et al. have discovered that if the MGMT promoter responsible for MGMT expression is methylated, then patients have higher chances of survival (55) (46)., Studies have shown that the MGMT signaling pathway also plays a role in the TMZ resistance of glioma tumor cells (53). A combination of interferon-α (IFN-α) and antiepileptic drug levetiracetam (LEV) along with TMZ has shown to cause a potential decrease in the proliferation of glioma cells as per the *in vivo* study of subcutaneous xenografts and orthotopic xenografts in a mouse model (56).

### 4.2 Disrupted mismatch repair pathway

The cytotoxicity of TMZ is mediated mainly by a DNA-MMR mechanism on the O6-MeG base. MMR proteins are active role players in determining the efficacy of TMZ, as their deficiency or a mutation in the MMR pathway can create TMZ resistance during treatment through interruptions in the breaking of DNA-mispaired chains (11). During the MMR mechanism, thymine attempts to bind O6-MeG through the “futile DNA repair cycle” in order to fix the mismatch repair, leading to cell-cycle arrest and apoptosis (**Figure 5B**). The presence of MMR-deficient cells in the tumor promotes the development of resistance against standard chemotherapeutic agents such as TMZ, procarbazine, and cisplatin. Low-abundance MMR proteins such as MSH2 and MSH6 are correlated with increased TMZ resistance and tumor progression (57, 58).

### 4.3 Base excision repair

Base excision repair is a prevalent system to repair DNA in mammalian cells. It plays a significant role in the maintenance of genome integrity as it eliminates nucleobases with small modifications from the cell cycle. The BER mechanism recruits glycosylase, endonuclease, polymerase, and DNA ligase to repair single-nucleotide modifications such as methylation, oxidation, deamination, and single-strand breaks (SSBs) (52). Methylation adducts formed at N3-MeA and N7-MeG are very abundant (>90%); however, their cytotoxic effects are very limited owing to rapid detection and repair by BER mechanisms (59). When GBM cells treated by TMZ are marked by methylation at N3-MeA and N7-MeG, BER will detect the wrong paired nucleosides and fix the DNA chain using a lesion-specific DNA glycosylase during replication cycles (**Figure 5D**) (60). After the repair process, even though GBM cells were treated with TMZ, they lost N3-MeA and N7-MeG so the MMR system cannot detect any mistake in the DNA chain and cells will stay alive. Poly (ADP-ribose) (PAR) polymerase (PARP) catalyzes the synthesis of PAR. PARP-1 and PARP-2 recognize SSB produced by alkylating agents, oxidative stress, radiation, etc. However, PARP-1 is linked with the BER mechanism as a part of DNA damage response (DDR) (61, 62). Hence, several studies and clinical trials have been conducted to investigate the efficiency of PARP-1 inhibitors. However, due to the limitations of the blood–brain barrier (BBB) and heterogenous tumor response, this remains a clinical challenge (63).

### 4.4 GBM stem cell markers

Morphologically, GBM tumor masses possess heterogenous cells and contain subcell populations with the capacity of self-renewal and tumorigenesis known as tumor-initiating cells (TICs) or GBM stem cells (GSCs) (64). GSCs are abnormal neural stem-like cells (NSCs) which contribute to the development of pathological heterogeneity in astroglial tumors. GSCs are involved in pathological gliomagenesis (65). GBMs composed of GSCs are capable of creating spheres known as “neurospheres” which can regenerate new spheres and contribute to tumorigenesis (22). In addition to causing abnormal cellular proliferation and tumorigenesis, the GSC subpopulation is also involved in resistance developed against chemo- and radiotherapies (66).

As TMZ is still a standard key chemotherapy for GBM treatment, TMZ resistance makes GBM non-curable. This calls for the study of signature molecules to explore alternative options for adjuvant therapies (67). Reported GSC markers are CD133, SSEA-1, nestin, NANOG, SALL4, STAT3, SOX2, c-Myc, Olig2, Bmi1, CD44, L1CAM, SOX3, integrin-α6, and KLF4 (**Figure 5E**) (68–71),^,,^ Fundamental research on GSCs and associated molecular markers demonstrates that GSCs are capable of tumorigenesis, self-renewal, differentiation, and resistance to chemo- and radiotherapies (2, 69, 72–74). *In vitro* studies indicate the potential of GSCs in forming tumor spheroids. In addition, *in vivo* xenotransplantation of GSCs into immunocompromised mice *via* subcutaneous cell injection reforms tumors with identical histological characteristics (75). GCSs treated with various common antineoplastic agents (etoposide, camptothecin, cisplatin, TMZ, doxorubicin, vincristine, etc.) exhibited significant resistance against chemotherapies and in some cases recovery followed by cell survival and proliferation (75, 76).

### 4.5 Autophagy

Autophagy is an intracellular degradation process, which disintegrates the cytoplasmic components using lysosomal machineries (77). In other words, a cell can self-digest its own cellular components in the lysosome (78). Autophagy becomes important during starvation and cellular stress whereby organelles, cytoplasm, and cellular proteins are engulfed, consumed, and recycled to maintain normal physiological activity in the body (79). Based on physiological functions and modes of transportation of cytoplasmic components to lysosomes, three forms of autophagy are defined: chaperone-mediated autophagy, microautophagy, and macroautophagy (normally known as autophagy) (**Figure 5F**) (78). Autophagy is generally seen as important in cancer prevention. Controversially, it has been suggested that under neoplastic conditions autophagy can promote cell survival (80). Defects in the autophagy mechanism can result in cancer and be associated with oxidative stress, activation of DNA damage response, tumorigenesis, and genome instability (81). Normal physiological autophagy events exert a cytoprotective effect by degrading misfolded proteins, damaged organelles, and reactive oxygen species. This results in regulation of aberrant mutations and ultimately cancer (82). Defects in autophagy genes or autophagy mechanisms can lead to neoplastic conditions such as allelic loss of the beclin1 tumor-suppressor gene in epithelial ovarian carcinoma (83). Autophagy keeps a paradoxal double role as it can both suppress and promote tumors (82, 84).

### 4.6 Epigenetic modifications

Epigenetic alteration is one of the several mechanisms of cancer drug resistance. The “epigenetic” term was coined by Conrad Waddington and defined as “the branch of biology which studies the causal interactions between genes and their products, which bring the phenotype into being” (85). With further research and more detailed understanding of the epigenetic mechanism, the definition of epigenetics has evolved. Recently, it has been defined as “heritable changes in gene expression without changing the DNA sequence” (86). The main epigenetic modifications are DNA methylation of cytosine, acetylation/deacetylation of histone proteins, and nucleosome positioning. In DNA methylation, the methyl group is attached to the 5′ position of cytosine on the CpG island, whereas the histone modification involves methylation, acetylation, reader mutations, and phosphorylation of histone proteins (87). Understanding the molecular mechanism of TMZ resistance at the epigenetic level can provide a novel approach to identify new targets that can restore the efficiency of TMZ. A recent study on long non-coding RNAs (lncRNAs) indicates that LncRNA SOX2OT (sex determining region Y-box 2 overlapping transcript) elevates SOX2 expression and is associated with tumor growth and poor prognosis (88). Aberrant expression of lncRNAs is associated with therapy-resistant glioma or GBM. Oncogenic lncRNAs such as MALAT1, NEAT1, H19, MIAT, UCA, HIF1A-AS2, XIST, and HOTAIR are significantly influenced by chemotherapeutic agent TMZ (89, 90). Developing inhibitors against such epigenetic alterations could be beneficial in terms of improving the clinical advantages provided by standard therapies. Histone acetylation and deacetylation are two major events involved in histone-related epigenetic alterations. Histone acetyl transferase (HAT) adds an acetyl group to histone, whereas histone deacetylase (HDAC) removes an acetyl group (91). Such epigenetic modifications support the acquisition of adaptive TMZ resistance during treatment. Specific genes emerge to extend cell survival and proliferation (92). Epigenetic modifications such as DNA methylation, histone acetylation/deacetylation, chromatic remodeling, and lncRNAs have been explained in GBM (**Figure 5C**) (93). To obtain clinical benefits from a TMZ chemotherapeutic regime, many clinical studies are in development to test and develop histone deacetylation inhibitors (93). To target epigenetic mechanisms, inhibitors have been designed and are presently under clinical trial investigation (phase I/II/II). Their use would be in a combined therapy along with TMZ as the primary treatment (93).

### 4.7 MicroRNAs

MicroRNAs (miRNAs) are single-stranded, non-coding regulatory RNAs that contain 22–25 nucleotide bases (94). They are well studied for their involvement in neoplasticity of GBM tumors (95). MicroRNAs are thought to act as posttranscriptional regulators in gene expression and in cell proliferation, angiogenesis, and generation of CSCs (96). MicroRNAs are not only capable of driving the neoplastic behavior of GBM but also key in the acquisition of TMZ resistance. For example, miR-21, miR-195, miR-455-3p, miR-10a, miR-181b, and miR-181c are reported to be heavily involved in TMZ resistance (**Figure 5G**) (97). Targeting miRNAs using inhibitors can restore the cytotoxicity of TMZ, such as by transfecting overexpressing miR-21 GBM cells with miR-21hibitors, which reveals resensitization of TMZ-resistant cells. MicroRNAs are receiving attention as biomarkers and as potential therapeutics that target GBM (97).

### 4.8 Extracellular vesicles

Extracellular vesicles are heterogeneous, vesicular, bilayer lipid structures of varying sizes (50 to 1000 nm) released by all cells. From those cells, they carry a molecular cargo such as lipids, proteins, DNA, mRNA, and miRNAs (67). Common extracellular vesicles (EVs) such as exosomes, microvesicles, oncosomes, and microparticles are involved in various cancers including breast, prostate, GBM, gastric, and colorectal (**Figure 5H**). Proteomics analysis of EVs shows that proteins involved in the cell adhesion pathway are involved during TMZ treatment and responsible for drug resistance (98). The mediator role of EVs in intercellular communication in tumor microenvironments affects chemotherapy treatments. Hypoxia is associated with tumorigenesis and induces chemoresistance in GBM. Particularly, GCS-derived EVs have shown to increase TMZ resistance significantly through miR-30b-3p (99). The capability of EVs to transfer lipids and proteins to tumor microenvironments promotes invasion, angiogenesis, and resistance against anticancer drugs (100). Despite limited knowledge of the mechanisms of secretion and anticancer promotion by EVs, additional research work is needed to clearly understand the cargo capacity of EVs.

## 5 Molecular bases for CNS tumors, GBM classification, and TMZ resistance

One topic of interest is the genetic background of GBM and TMZ resistance. The 2016 classification of CNS tumors is based on an integrated approach for diagnosis to achieve reproducibility, clinicopathological prediction, and treatment planning (29). According to this integrated method, the disease should be diagnosed based on histology and molecular information to justify the types or specific subtypes of tumors, including GBM. Mutations in markers either are early indicators of tumorigenesis or represent the specific progress of cancer such as in the case of high- or low-grade glioma tumors (29). The identification and assessment of such markers determine the clinical outcomes and specifically if the first line of treatment of TMZ would be beneficial to the patients or not and if additional adjuvant therapies are needed. Characteristic molecular markers for CNS tumors and GBM are described below in correlation with TMZ treatment outcomes.

### 5.1 Isocitrate dehydrogenases

**Figure 6:**
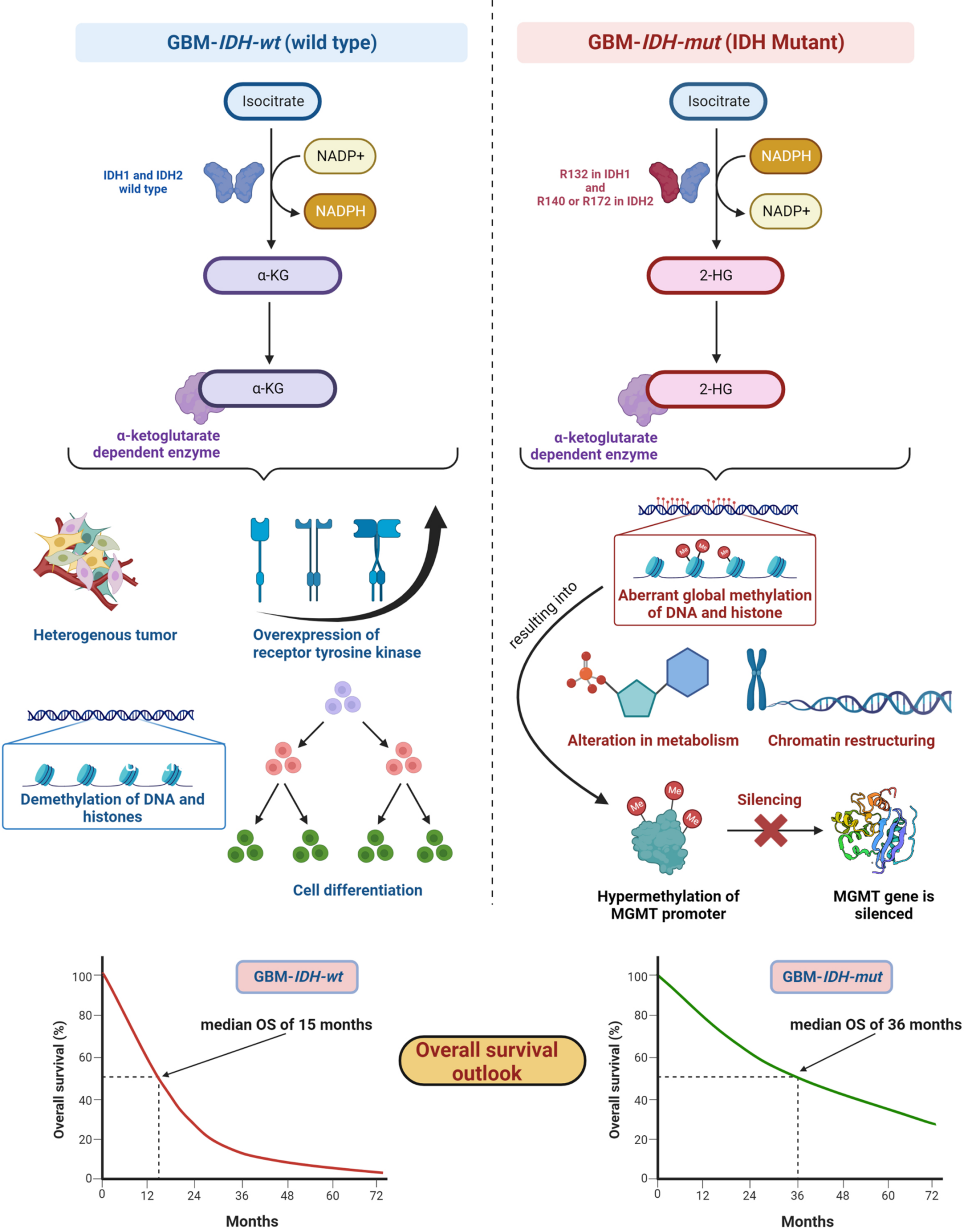
IDH status in GBM (1) GBM-IDH-wt and (2) GBM-IDH-mut: IDH-1 is localized in cytoplasm whereas IDH-2 is mitochondrial. In normal conditions, IDH1 and IDH2 catalyze substrate isocitrate to α-KG. Mutation on arginine on 132 to histidine (R132H) is most frequent mutation of IDH-1 in GBM; however, R140 or R172 is a common mutation in IDH-2 (106). IDH-mutation generates 2-HG as an oncometabolite. 2-HG create intense effect on cellular system *via* alteration in metabolism, chromatin restructuring, and aberrant global methylation of DNA and histone, which further hypermethylates MGMT promoter and consequently silences MGMT gene resulting into extended OS compared with IDH wt-type. IDH-wt results into heterogeneous tumor formation, alteration in receptor tyrosine kinases (EGFR and PDGFRA), demethylation of DNA and histones, and cell differentiation (107). Median OS for IDH-GBM-mut is 36 months however it is only 15 months in case of GBM-IDH-wt.

IDH, metabolic enzymes, are categorized as IDH1, IDH2, and IDH3. The enzyme IDH1 is localized in the cytoplasm and in the peroxisomes (101). It is involved in lipid metabolism and glucose sensing. Enzymes IDH2 and IDH3 are present in mitochondria and involved in the Krebs cycle (102). As key functions, all IDHs catalyze oxidative decarboxylation of isocitrate and transform it into α-ketoglutarate (αKG) in the citric acid cycle (103). Enzymes IDH1 and IDH2 are associated with unique tumor-cell metabolism. For the first time, Parsons et al. discovered mutations in IDH in human GBM (104, 105). As per the 2016 WHO CNS classification, GBMs are of two types based on IDH status: (1) GBM-*IDH-wt* (wild type) and (2) GBM-*IDH-mut* (mutant) (28). Details about the mechanism, characteristics, and impact on median overall survival (OS) for GBM-*IDH-wt* and GBM-*IDH-mut* are shown in **Figure 6**. GBM patients with IDH-wt represent poor OS (around 15 months) in comparison with those with IDH-mut (around 36 months) (108). In another study, the same was reported, in that glioma patients with IDH1 or IDH2 mutations had a better outcome compared with patients with *IDH-wt* where no IDH mutation was observed (105). IDH1 and IDH2 mutations are commonly found in low-grade glioma (70% of WHO grade 2 and 3 astrocytomas and oligodendrogliomas) and secondary GBM (which is developed from lower-grade gliomas). Primary GBMs rarely show the IDH mutations. IDH1 mutation is correlated with MGMT promoter methylation (109), and patients with the hypermethylated MGMT promoter are sensitized to TMZ treatment (109).

GBM-*IDH-wt* represents the characteristic of interpatient differences, heterogeneous tumors, overexpression of receptor tyrosine kinase, normal methylation of DNA and histones, and significant cell differentiation (107). Due to the larger proliferative and invasive capacity of *IDH-wt* type tumors than *IDH-mut*, the median OS of GBM-*IDH-wt* is much shorter compared with GBM-*IDH-mut*. *IDH-mut* decreases the normal catalytic activity and produces lower amounts of α-ketoglutarate (α-KG) and NADPH. Lowering the amount of α-KG results in the generation of 2-hydroxyglutarate (2-HG), an oncometabolite, using NADPH (109, 110). The accumulation of 2-HG increases the local and genome-wide methylation pattern. Hence, hypermethylation of the MGMT promoter silences the overall MGMT expression in patients, leading to increased toxicity of TMZ (105, 111). Additionally, IDH1 or IDH2 mutations were found in colorectal cancer, prostate cancer, thyroid carcinoma, melanoma, and acute myeloid leukemia (112).

Fundamentally, the detection of IDH mutations in GBM denotes an early episode of gliomagenesis and the presence or absence of such mutations determines the disease progression and therapeutic outcomes (113, 114). Considering these facts, IDH mutants or wild-type profiles have become reliable diagnostic and prognostic markers in GBM (115). The GBM-*IDH-wt* status indicates the shorter median OS of around 15 months. The 2016 CNS WHO classification, which incorporated molecular markers as part of layered information for an integrated diagnostic approach, was a vital step forward. Detection of IDH status in GBM with the other markers became an essential part of standard diagnosis of CNS tumors (29).

### 5.2 The biomarker O^6^-methylguanine-DNA methyltransferase

Higher MGMT expression levels correspond to poor clinical outcome for patients; hence, MGMT levels are an important factor in determining the therapeutic benefits of TMZ. MGMT protein levels vary according to organs, with highest levels (liver) and relatively low levels (brain). Usually, MGMT levels in tumors are higher than in the healthy tissue of origin. The CpG islands of MGMT genes are not methylated in normal tissues (46). However, methyl-CpG-binding proteins alter the chromatin structures and prevent the binding of transcription factors, resulting in silencing of the MGMT gene. It has been shown that TMZ chemotherapy is more efficient when lower levels of MGMT protein are present. In such cases, patients have longer OS rates and progression-free survival (PSF) rates (116). Glioma patients with MGMT CpG promoter methylation have shown prolonged PFS and OS (117, 118). In the MGMT gene, methylation of the CpG island inhibits transcription of the gene, and cell lines with the methylated promoter of MGMT cannot repair alkylation in O6-methylguanine.

Efforts have been made to improve the antitumor effects of TMZ including development of pseudosubstrates, interfering RNA (RNAi), viral proteins, and many other agents. It has been revealed that cell exposure to alkylating agents induces nuclear factor-kappa B (NF-κB) activation, which increases MGMT expression.

### 5.3 BRAF

The v-raf murine sarcoma viral oncogene homolog B1 (BRAF) is associated with RAF serine/threonine protein kinases. The three RAF family proteins are RAF1/CRAF, BRAF, and ARAF. BRAF mutations were identified in various cancers including 59% in melanomas, 18% in colorectal cancers, 11% in gliomas, and 14% in liver cancers (119). With regard to primary brain tumors, V600E mutations and KIAA1549–BRAF fusions are the most commonly observed type of mutations. Firstly, BRAF-V600E mutations (valine substituted with glutamic acid at position 600) are present in gliomas including pleomorphic xanthoastrocytoma, ganglioglioma, pilocytic astrocytoma, low-grade gliomas and pediatric GBM. Secondly, KIAA1549-BRAF fusion is the most common BRAF alteration in pilocytic astrocytoma. This is also commonly found in malignant melanomas, papillary thyroid carcinomas, and colorectal carcinomas (120).

The BRAF protein participates in the cascade of the Ras-Raf-MEK-extracellular signal-regulated kinase (ERK) or mitogen-activated protein kinase (MAPK)/ERK signaling pathway, which affects cell division and cellular differentiation. Gliomas along with BRAF mutations also show additional alterations in tumor protein P53 (TP53), TERTp, CDKN2A/B, and PTEN and favor the response to BRAF and/or MEK inhibitors (121). Various RAF inhibitors (Vemurafenib™, Dabrafenib™, Encorafenib™) and MEK inhibitors (Cobimetinib™, Trametinib™, Binimetinib™) have been approved to improve clinical output in the treatment of various cancers. However, if CNS tumors show heterogeneity, resistance development against inhibitors does not provide guaranteed therapeutic benefits from the treatments (120).

### 5.4 EGFR and PTEN

The epidermal growth factor receptor (EGFR) is a cell surface receptor and tyrosine kinase, which shows amplification and/or mutation in several types of cancers. EGFR has been identified as a contributor to tumor growth (122). Four members of the EGFR family (ErbB1 (EGFR), ErbB2, ErbB3, ErbB4) are known to be actively involved in cell division, differentiation, and apoptosis (123). EGFR overexpression and gene alteration are regularly observed in rapidly developing primary GBM and do not signify the existence of less malignant precursor lesions. It has been proven that EGFR signaling is often interrupted by frequent events related to EGFR gene amplification (protein overexpression) and mutation in the neoplasm (124). Tumorigenic cancer stem cells (CSCs) are well recognized for their role in aggressive disease progression and recurrence (74). The heterogeneity of CNS tumors is due to various factors including the generation of CSC as the disease progresses. CSC are capable of self-renewal, constant spreading in surrounding areas including healthy cells or tissues, and tumorigenesis upon secondary transplantation (74, 125). It has been revealed that tumorigenic glioma stem cells populations show chemo- and radio-resistance after treatment in glioma (74, 126, 127). Alterations in EGFR oncogenes are observed in half of the primary GBMs and result in resistance against chemo- and radiation therapies. Recently, efforts have been made to fight resistance by targeting EGFR and DNA using “combi-molecules” (designed to aim at two targets in cancer cells) (66). Various therapeutics such as monoclonal antibodies (Cetuximab™, Nimotuzumab™), inhibitors (Gefitinib™, Erlotinib™) are known to trigger different EGFR signaling pathways to enhance the survival of cancer cells and to develop resistance against therapeutic agents (128). Cellular stress introduced by chemotherapy has shown similar effects. Specifically, the EGFR variant III (EGFRvIII) mutation is the most common in GBM and is associated with tumorigenicity and poor prognosis in GBM patients (129). The exact mechanism for generating EGFRvIII is still unclear; however, its presence increases cell proliferation (123). For increased overall survival of patients, various inhibitors and adjuvants have been developed, although with limited success. Several small-molecule- and antibody-based therapies have been implemented, but development of drug resistance followed by secondary resistance against these inhibitors has resulted in poor clinical outcomes (130, 131).

The deletion of phosphatase and tensin homologs on chromosome 10 (PTEN) results in a tumor-suppressor gene, which regulates cellular growth, proliferation, survival, apoptosis, metabolism, and cell migration and also carries functional roles in the nervous system. PTEN mutations have been observed in older over-survived patients; however, there is no strong connection with over-survival (132).

From a clinical standpoint, there are two types of GBM: primary and secondary. Primary GBM occurs more often (~90% of cases) with tumors that develop *de novo*, often in older patients and without any history of less malignant forms of precursor lesions. Secondary GBM develops gradually from lower-grade diffuse astrocytoma or anaplastic astrocytoma in younger patients at diagnosis. From a genetics perspective, primary GBM manifests EGFR amplification, PTEN mutation, and the entire loss of chromosome 10, whereas secondary GBM shows frequent mutations in the TP53 gene (encodes tumor-suppressor protein p53) with longer overall survival (133). However, recent studies indicate that TP53 mutations are crucial roles players in developing radio-resistance and are associated with poor survival in specific CNS-related neoplasms (134, 135).

### 5.5 Alpha thalassemia X-linked intellectual disability and tumor protein P53

Alpha thalassemia X-linked intellectual disability (ATRX) belongs to the SWI/SNF2 (SWItch/sucrose non-fermentable) family of chromatin remodeling proteins. The ATRX gene was first found in patients with the alpha thalassemia X-linked intellectual disability syndrome (136). A mutation in ATRX leads to the “alternative lengthening of telomeres” (ALT) phenotype and to genomic destabilization (137).

The wild-type tumor protein TP53 plays an inhibitory role against cellular growth when DNA damage occurs. A mutation in the TP53 gene changes the protein function, leading to failure in preventing cellular replication and promoting proliferation of neoplastic astrocytoma (138).

Four molecular features that differentiate diffuse astrocytoma and oligodendroglioma are IDH wild type/mutants, TP53 mutations, ATRX loss, and 1p19q codeletion. Diffuse astrocytomas are either IDH mutants with ATRX loss and TP53 mutations, or IDH wild type. IDH mutants with 1p/19q codeletion are characterized as oligodendrogliomas (139). The ATRX mutation shows rapid progression of glioma tumors *vs*. wild type and hence has therapeutic potential (140).

## 6 LC-MS-based proteomics studies in human GBM and TMZ resistance

**Figure 7:**
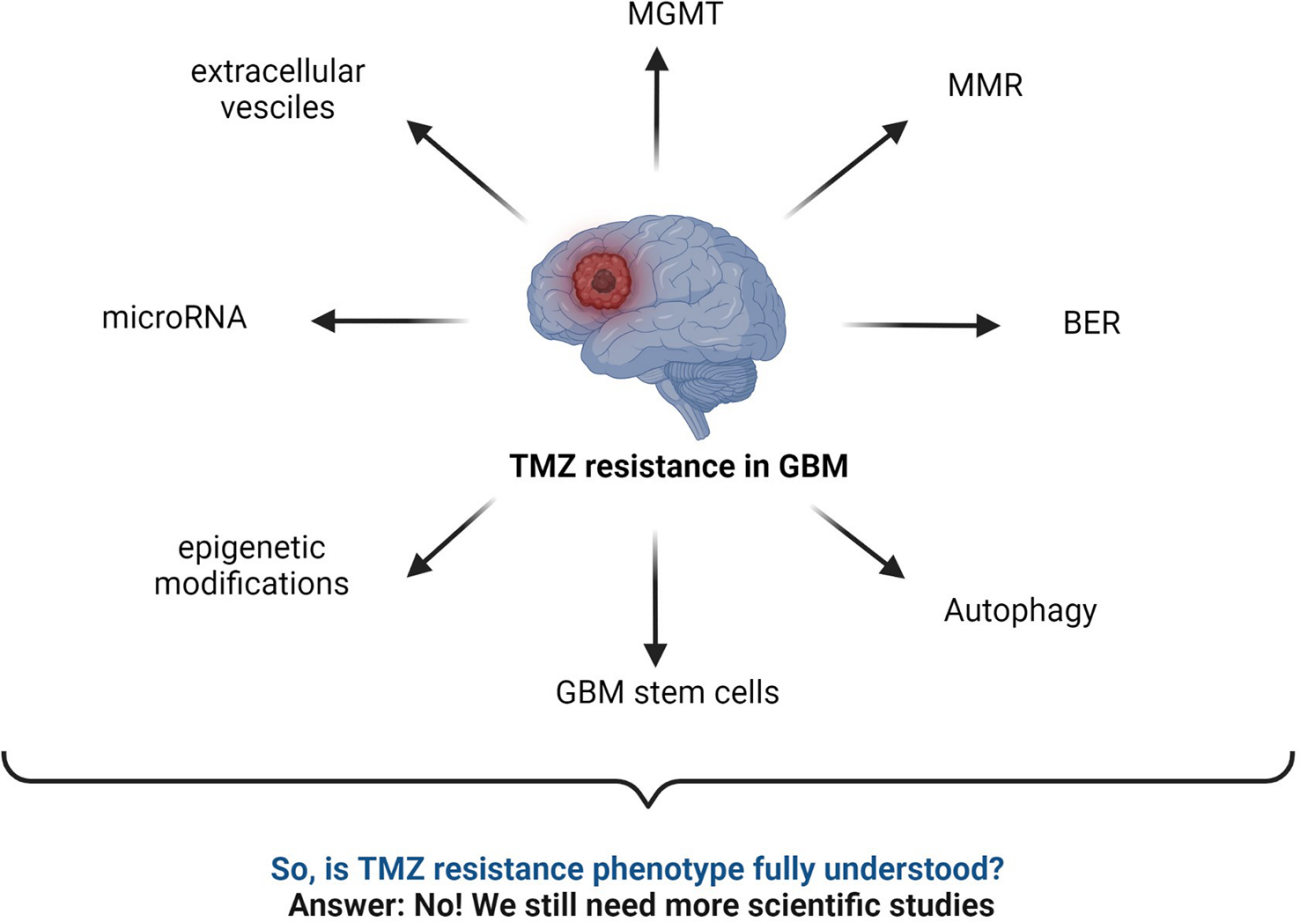
Summary of commonly known mechanisms for TMZ resistance in GBM. MGMT status, BER mechanism, interrupted MMR mechanism, autophagy, microRNA, GBM stem cells, epigenetic modifications, extracellular vesicles, etc., are major responsible factors reported for TMZ resistance (2, 43, 52, 59, 141–143).

Several TMZ resistance mechanisms have been discussed in the literature. Still, the TMZ-resistance hurdle has proven to be a significant challenge due to the complex nature of GBM (**Figure 7**). The TMZ resistance mechanism is very complex, and studying cell signaling pathways and protein–protein interactions (PPI) can provide crucial information on the matter. Finding early signature biomarkers can also be key in adjusting the treatment strategy (144). OMICS research with high-throughput technologies such as next-generation DNA sequencing, proteomics by liquid chromatography-mass spectrometry (LC-MS/MS), metabolomics, and genomics has revealed its potential for helping to understand various forms of cancers at the molecular level (145). In particular, LC-MS/MS-based proteomics has widely served the OMICS purpose in cancer cell biology and drug resistance mechanisms.

Despite remarkable advances in the molecular biology towards understanding neoplastic phenotypes, the therapeutic success rate is still low for many types of cancers. The limitations of clinical benefits from chemotherapies in the treatment of GBM indicate that there is still a considerable amount of research necessary to gain more understanding of drug resistance mechanisms. Mutations are not fully understood yet, and alternative pathways of chemotherapeutic resistance are activated when specific proteins are targeted during treatment. Proteomic studies offer insightful approaches to better understand the downstream effects of cancer-related genotypes (146).

This section discusses possible LC-MS-based workflows to study cellular proteomics with the goal of better understanding resistance phenotypes in GBM. For years, proteomics has gained a very important role in glioma research, both for the investigation of disease pathobiology and for the development of efficient therapeutics against GBM and other forms of tumors. In addition, glioma proteomics can potentially identify biomarkers, which is very helpful for diagnosis, treatment decisions, prognosis, and assessment of treatment response (147). Not only the primary (amino acid sequence) and secondary structures can account for the function of proteins, but posttranslational modifications such as glycosylation, acetylation, and phosphorylation can directly affect how proteins function. Advanced high-throughput LC-MS-based proteomics is one of the indispensable techniques in cancer biomarker discovery. Recent advances in chromatography and MS technology have revealed the remarkable capabilities of high sensitivity and high-resolution multiplexed quantitation. LC-MS/MS-based proteomics is used to identify differences in protein expression and lends itself to pathway analysis in various cancerous tumors with radio- and chemoresistance phenotypes (148).

**Figure 8:**
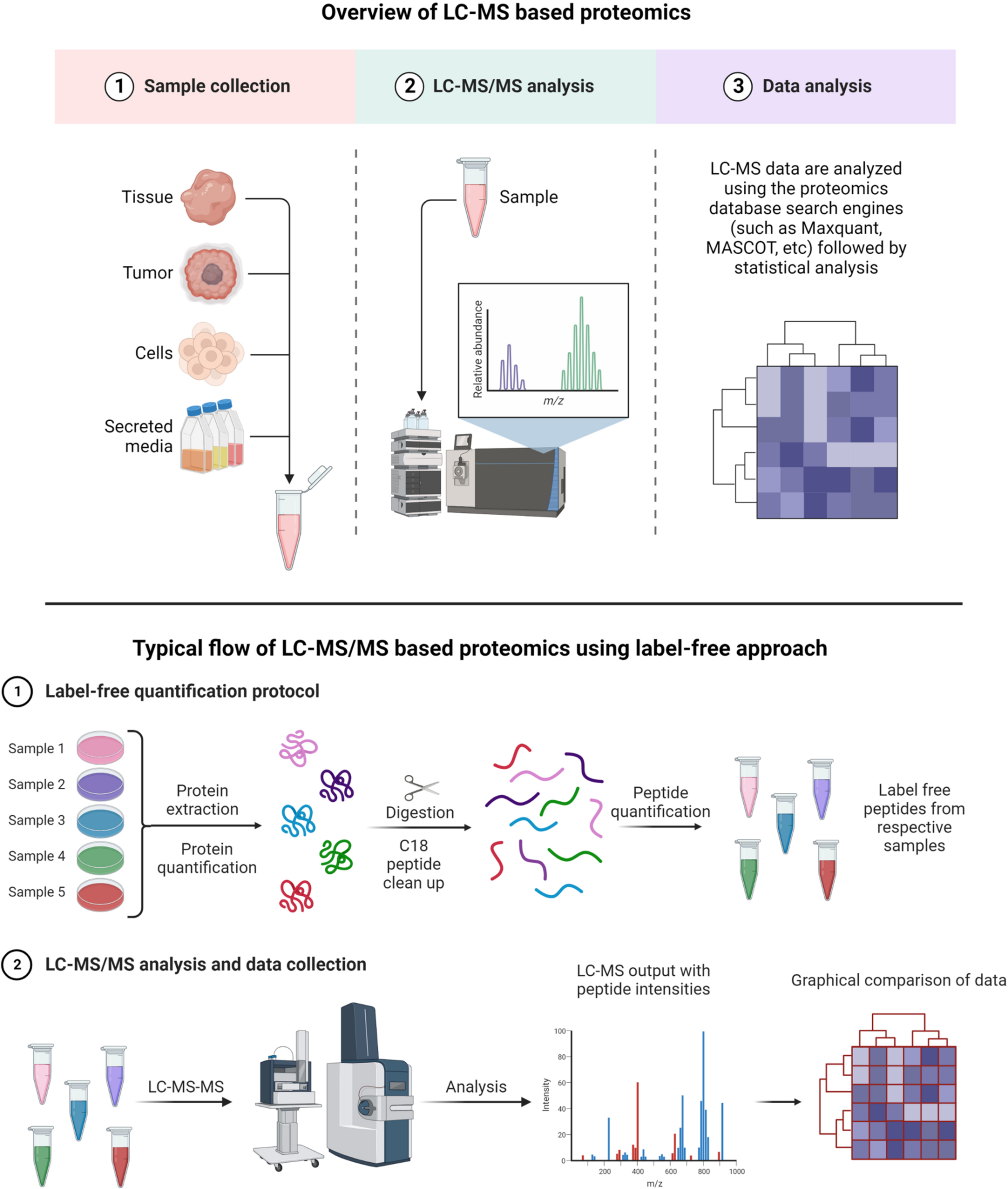
Example of proteomics workflows. Top figure: Biological samples such as tumors, cells, tissues, and secreted media are processed to extract the proteins. Extracted proteins are digested and analyzed by LC-MS/MS. Raw files generated from LC-MS/MS are used in a proteomics database search engine for protein identification and quantitation. Bottom figure: tandem mass tag (TMT) labeling is used to perform multiplex analysis for simultaneous relative quantification of proteins and for identification (149, 150).

**Figure 9:**
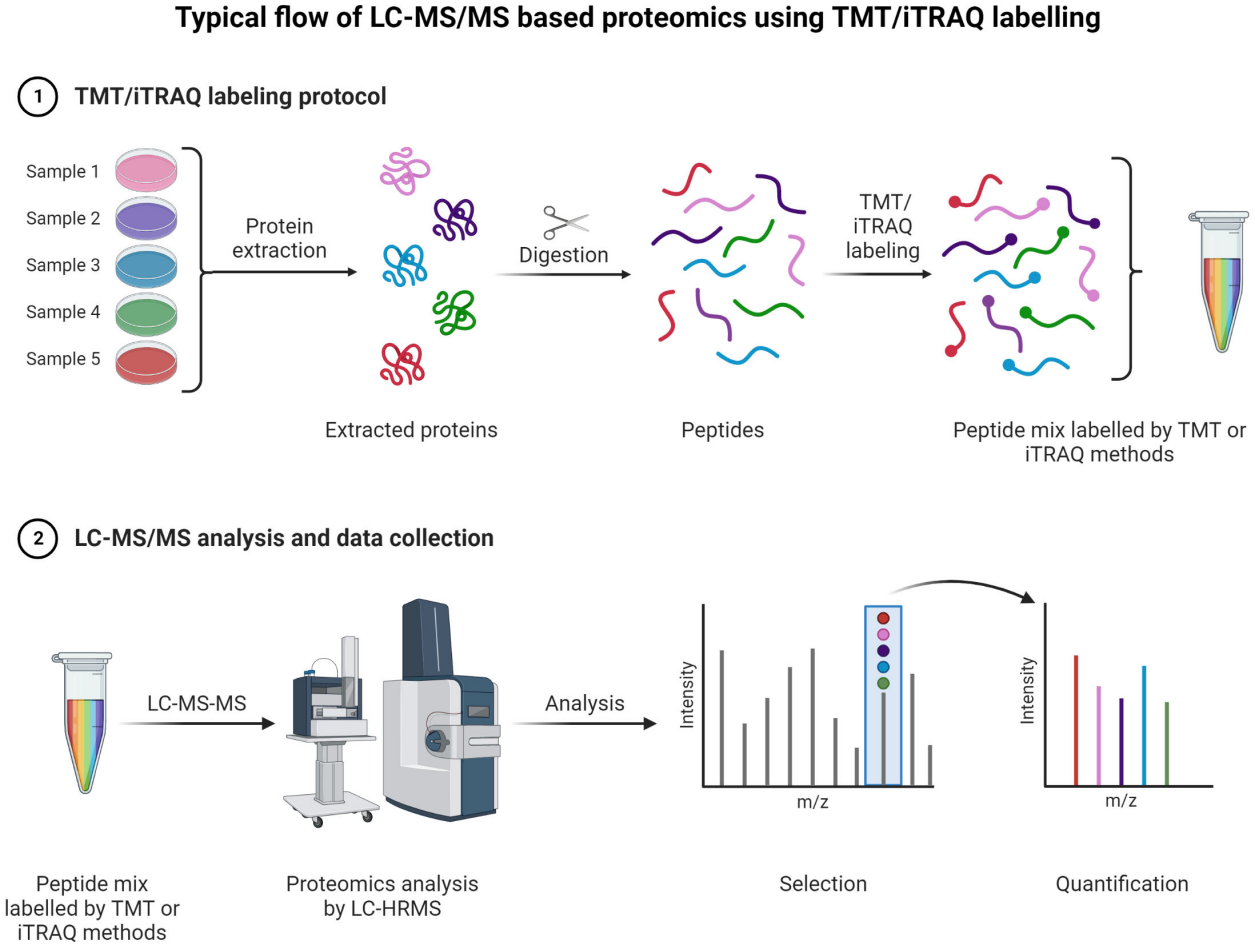
TMT or iTRAQ labeling is used to perform multiplexed (up to 18-plex) analysis for simultaneous quantification of proteins and identification (149, 150) (151, 152). Proteins are extracted and digested with respective enzyme(s). The peptides are labeled with TMT or iTRAQ multiplexed (up to 18-plex) reagents and mixed pool of TMT or iTRAQ labeled peptides are analyzed LC-MS (153–155).

A typical LC-MS proteomics workflow includes selection of sample type (cancerous cells, tumor, tissue, and secreted media), extraction of proteins, LC-MS/MS analysis, database search, and interpretation (**Figure 8**-top). Accurate and efficient quantification of proteins using the labeling of peptides is a very popular approach in LC-MS-based proteomics, e.g., label-free (**Figure 8**-bottom), tandem mass tag (TMT) labeling, and iTRAQ (isobaric tagging for relative and absolute quantification) (**Figure 9**). These methods are used to perform multiplexed and simultaneous quantification of proteins and further identification.

Label-free quantification has gained popularity in large-scale proteomics and biomarker discovery, as there is no need for isotopic labeling and thus low costs are associated with such experiments (156). The key advantage of the label-free method is a wide-range proteome span for identification and quantification of proteins in high and low abundance (157, 158).

TMT and iTRAQ are labeling techniques that allow MS/MS quantification along with enhanced sensitivity for MS. They are very useful for relative and absolute quantification (159). The key advantage of TMT is allowing for multiple tagging and thus reducing overall LC-MS/MS analysis time as multiplex mixed samples can be analyzed simultaneously (160). Peptides are labeled with multiplex reagents (up to 16-plex) that all yield the same molecular ion m/z value for a particular peptide. Upon MS/MS dissociation, fragment ion masses are unique with respect to the original label. Relative abundances can be determined with ease using this method for particular peptides from proteins with different levels of expression. With iTRAQ, the principle is the same with the possibility of analyzing 4-plex or 8-plex samples.

There are many cancer-related questions that cannot be answered using genomics data alone. Hence, The Cancer Genome Atlas (TCGA) aims to get full insight into cancer at the protein level (161). The capabilities of MS technology are continuously evolving, with improved protein identification at lower detection limits for complex matrix systems such as tissues, cells, and various forms of biological fluids (serum, plasma, urine, etc.). MS is also capable of determining post-translational modification with great accuracy and of quantifying proteins in a robust and reliable manner. Proteomics is a tool of choice to establish links between genotype and protein function. The Clinical Proteomic Technology Assessment for Cancer (CPTAC) aims to understand the molecular aspects of cancer by studying proteins resulting from alterations found in genomics data of various cancers (162). PTAC utilizes two key methods: “Targeting Genome to Proteome” (Targeting G2P) and “Mapping Proteome to Genome” (Mapping P2G) to advance further understanding of various types of cancer. The site of cBioPortal provides open-source multidimensional genomics data for various cancers (163). In order to create high-quality cancer proteomics datasets, a global collaboration is needed among clinical oncologists and scientists of all relevant fields. CPTAC has extensive data sets related to breast, colon, and ovarian cancers from TCGA. A recently published article summarizes an integrative CPTAC study on GBM using proteogenomics and metabolomics data generated using 10 multidimensional types of analyses: whole-genome sequencing (WGS), whole-exome sequencing (WES), RNA sequencing (RNA-seq), microRNA-seq (miRNA-seq), single-nucleus RNA sequencing (snRNA-seq), DNA methylation arrays, proteomics, phosphoproteomics, acetylomics, lipidomics, and metabolomics (164). This informative study suggests to conduct further research to reveal the complexity of GBM in order to provide stratification of various tumor types for efficient clinical management. These complexities of GBM are attributed to the heterogenous nature of CNS tumors.

Despite rigorous research on molecular characterization of GBM, the key challenge in understanding the development of drug resistance remains unchanged, whether it concerns intrinsic or acquired drug resistance. During treatment, multidrug resistance (MDR), defined as resistance from neoplastic cells against a wide variety of chemotherapeutic agents, remains an unresolved problem in GBM (13). Some accepted MDR events include for instance increased ability to repair DNA, genetic factors, enhanced efflux of drugs, growth factors, and metabolism of xenobiotics (165). The complexity of MDR extends well beyond these mechanisms, and understanding the exact processes taking place is necessary for the development of strategies for personalized treatments.

Over the last two decades, genomics has contributed significantly to reveal genetic alterations and signaling pathways associated with various cancers (161, 166). Proteomics, or the study of proteins from specific biological systems, has the potential to provide insight into the expression levels of proteins, their regulatory functions, de-/activation, PPI, cellular signaling, and PTMs.

Cellular heterogeneity in tumors is a main feature in GBM. Several studies have been conducted to understand the driving force behind such complex tumor microenvironments, and LC-MS-based proteomics has been the most effective approach. Proteomic approaches other than LC-MS have been utilized to investigate the biology of GBM cell lines, tumors, plasma, and serum samples. These methods involve 2D gel electrophoresis, matrix-assisted laser desorption/ionization mass spectrometry (MALDI-MS), and electrospray ionization-MS, among other techniques. **Table 1** gives examples of recent literature exploring analysis of human GBM specimens of the types mentioned above.

**Table 1:** Selective list of proteomics studies conducted (from year 2000 to date) on human GBM specimens to understand pathophysiology and cellular biology. .

Year period published (2000-to date)	Title of study	Types of specimens	Histopathology of specimens and/or number of samples	Specific experiments/test and/or instrumentations for LC-MS	Methodology or treatments of raw data	Summary of results	Conclusion
2014	Identification of Novel Tumor-Associated Cell Surface Sialoglycoproteins in Human Glioblastoma Tumors Using Quantitative Proteomics (167)	Human GBM tumors	*GBM tumor cells from four male patients *Neural progenitor cells (NPCs) and astrocytes from human fetal brain tissue	*Labeling and enrichment of sialoglycoproteins *Flow cytometry (cell surface proteins), confocal microscopy *RT-PCR (gene expression analysis) *Tryptic digestion and nanoLC-MS/MS analysis)	*Protein identification (MASCOT 2.1) *Quantitation by inhouse label free software and differential Fourier transform analysis) *Protein annotation (subcellular localization and functions) using Ingenuity Pathway Analysis Knowledge Base (http://ingenuity.com/), Human Protein Reference Database, UniProtKB/Swiss-Prot protein knowledgebase, Gene Ontology (http://geneontology.org/), and PANTHER (http://www.pantherdb.org/) *Gene expression (BioGPS, http://biogps.gnf.org/) and from the Gene Expression Atlas database (http://www.ebi.ac.uk/gxa/). Gene expression data from ArrayExpress archive (http://www.ebi.ac.uk/arrayexpress/) *Protein expression (Human Protein Atlas (HPA) portal (http://www.proteinatlas.org/)	*606 cell surface proteins (p-value <0.05) identified and quantified. *35 cell surface proteins overexpressed in GBM compared with astrocytes and NPCs *Gene expression analysis revealed 23 genes associated with GBM in accordance with glycoproteomic analysis. *19 out of 23 genes indicated correlation between mRNA expression and protein expression in GBM and astrocytes. *Known biomarkers for glioma biology: PTPRZ1, GPR56, TNC, IL13RA2, ICAM1, NCAM1, THBD, and NTRK2 *Novel markers for glioma biology: found: SLC1A3, CLU, LGALS3BP, ANGPTL2, CRYAB, and ITGB8	*Differentially expressed cell surface sialoglycoproteins from resected GBM tumors identified and quantified. *Proteomics and genetic expression analysis revealed already known and novel cell surface proteins linked with GBM phenotype. *Glycoproteomics/proteomics approach reveal therapeutic targets and lead to personalized oncotherapy. *Large-scale studies are required
2012	Investigation of serum proteome alterations in human glioblastoma multiforme (168)	Human serum samples	*N = 40 samples from GBM patients and n = 40 samples from healthy subjects	*2D electrophoresis (2DE) and 2D-DIGE *In-gel tryptic digestion and MALDI-MS, MS/MS analysis Immunoturbidimetric assay and Western blot analysis	*DeCyder 2D software analysis of 2D-DIGE gels *Protein identification based on UniProt accession number *Gene ontology terms for molecular functions, cellular components, and pathways	*2DE (n = 20) analysis revealed 10 differentially expressed protein spots *2D-DIGE (n = 8) identified a total of 136 differentially expressed spots. 82 proteins were upregulated and 54 down regulated *MALDI-MS/MS identified 27 differentially expressed proteins (21 upregulated and 6 downregulated) in GBM patients	*A few differentially expressed serum proteins were identified in GBM pathology.
2021	Extracellular Matrix Proteome Remodeling in Human Glioblastoma and Medulloblastoma (169)	Human GBM tumors	*Normal brain, 14 samples: 4 tumor samples of cerebellums and 10 isocortexes (5 frontal and 5 parietal) *Tumoral brain, 13 samples: 5 medulloblastomas (age 3 to 33 years, all male) and 8 GBM (age 29 to 60 years, three males: five females) samples	*Isolation of brain derived extracellular matrix (ECM) *Tryptic digestion *Nano-LC-MS/MS analysis	*Protein identification and label free quantification using MaxQuant (protein identification) *Gene ontology and PPI in STRING database ((https://string-db.org/) and Cystoscape (https://cytoscape.org/). Human protein atlas (https://www.proteinatlas.org/) *Tissue microarray and immunohistochemistry *Statistical analysis: data distribution using Kolmogorov–Smirnov test; expression levels by nonparametric Kruskal–Wallis and *post-hoc* Dunn tests among the groups; assessment of correlation between relative protein expression values—using nonparametric Spearman-rho correlation test.	*19 ECM proteins out of 84 were differentially expressed in medulloblastomas compared with cerebellum *28 ECM proteins out of 163 showed differential expression in GBM compared with isocortexes *40 ECM proteins were differentially expressed between GBM and medulloblastomas *GBM shows upregulation of FINC and BGH3 supporting invasive capabilities *TENA overexpression in GBM is associated with ECM stiffness *Downregulation of LAMA2 associates with proliferation and invasion in GBM *GBM aggressive growth is linked with overexpression of fibrinogen chains A, B, and C present in ECM *(S10AB) and annexin A2 (ANXA2) were overexpressed in GBM-ECM. S10A9 is secreted by tumor linked immune cells and mediates tumor aggressiveness.	*GBM ECM-proteomic profile shows increased ECM stiffness along with increased proteoglycan and glycoprotein diversity *GBM shows higher ECM turnover, invasion, and migration *Blood proteins found in GBM indicated abnormality in BBB
2012	LC-MS/MS Analysis of Differentially Expressed Glioblastoma Membrane Proteome Reveals Altered Calcium Signaling and Other Protein Groups of Regulatory Functions (170)	Human GBM and astrocytomas	*100 surgical biopsies collected, out of which 45 were astrocytomas and 22 out of 45 GBM *All were supratentorial GBM mostly derived from frontal lobe *Brain tissue obtained from temporal lobe epilepsy, young adult individuals (20–30 years) *Pooled control samples (n = 3) and GBM samples (n = 6) used to isolate microsomal fractions followed by iTRAQ labeling and LC-MS/MS	*Subcellular fractionation (to isolate the membrane) *Tryptic digestion and iTRAQ labeling *SCX column fractionation *nLC-ESI-MS/MS *Protein identification and quantitation *Immunohistochemistry and western blot analysis	*Biological function and cellular localizations—Human Protein Reference Database (http://www.hprd.org) and Gene Ontology *Pathway analysis by Ingenuity Pathway Knowledge Base *LC-MS raw data searched against NCBI RefSeq database (version 40) using Protein Discoverer (version 1.2) SEQUEST	*1,834 proteins identified *356 proteins showed ≥2-fold change, out of which 198 upregulated and 158 downregulated *Out of 356 differentially expressed proteins, 56% were membrane localized *Overexpression of EGFR CHI3L1, XRCC6, GOLIM4, SCARB2, ATL3 in GBM in GBM *EPB41L3, PALM2, NEGR1 were downregulated *Top three pathways: acute-phase response signaling, caveolar signaling, and calcium signaling were associated with cancer progression.	*The study highlights differential expression of membrane proteins in GBM pathogenesis
2003	Identification of differentially expressed proteins in human glioblastoma cell lines and tumors (171)	GBM cells	*Primary fetal human astrocytes (FHA) and glioma line U87MG (with low level EGFR) and U87MGΔEGFR (overexpressed EGFR)	*Cell growth in semi solid agar *2DE *In-gel tryptic digestion *MALDI-TOF analysis *Database search *Western blot and RT-PCR *ELISA (to determine the level of MMP2 secreted into cell media)	*Database search using MS-FIT proteomic tool UCSF server *MS-SEQ or NCBI Blast search for sequencing	*RT-PCR confirms wild-type EGFR and ΔEGFR in U87MG and U87MGΔEGFR, respectively *Overexpression of EGFR in U87MG cells increases proliferation and reduces apoptosis *52 proteins differentially expressed between U87MG and FHA *29 proteins overexpressed in U87MG compared with FHA *3 proteins U87MGΔEGFR compared with U87MG *Unique proteins found in FHA, related to development of clusters and found in neonatal brains *Differential expression in ECM components, cell surface molecules involved in adhesion and ECM hydrolyzing enzymes such as MMPs in U87MG indicates invasive potential	*Differential expression of proteins in FHA, U87MG, and U87MGΔEGFR provides further insight into how protein processing works and can lead to downstream effects on extracellular matrix and adhesion properties in GBM cells.
2014	Potential serum biomarkers for glioblastoma diagnostic assessed by proteomic approaches (172)	Serum samples	*35 patients (14 females and 21 males) and 30 healthy controls	*Serum preparation *SELDI-TOF MS *Biomarker delineation and sample fractionation *1D Page *Trypsin digestion *LC-MS/MS analysis *Validation by ELISA and Western blot assays	*MASCOT search	*3 biomarkers: CXCL4, S100A8, and S100A9 showed increased serum levels/tissue overexpression in glioblastoma compared with control *CXCL4 upregulation shows early tumor growth *S100A8 and S100A9 are overexpressed in various types of cancers including the current analysis in GBM. Linked with tumorigenesis.	Three proteins S100A8, S100A9, and CXCL4 were overexpressed and validation was performed by multiple techniques such as ELIS and Western blot in addition to SELDI-ToF MS and LC-MS/MS
2009	Proteomic Study of Human Glioblastoma Multiforme Tissue Employing Complementary Two-Dimensional Liquid Chromatography- and Mass Spectrometry-Based Approaches (173)	Human GBM tumors	*Tumor biopsy derived from 54-year male	*Histopathology revealed GBM *Extraction of protein from human GBM tissue *Semi top-down and bottom-up approach *Nanoflow IP-RP-HPLC-MALDI-MS/MS of peptides Protein identification and classification	*MASCOT version 2.2.03 *Scaffold Proteome software (version 2) was utilized to validate MS/MS based peptide and protein identifications obtained by Mascot *all MS/MS spectra were analyzed using both Mascot and X! Tandem (www.thegpm.org; version 2007.01.01.1) *Protein classification - Gene ontology	*2,660 proteins identified in GBM tumor samples and compared for biological functions.	Semi-top-down analysis of human GBM using IP-RP-HPLC-MS is a feasible approach for tumor tissue analysis as intact proteins can be eluted and targeted analysis can be performed. Semi-top-down can be a complement of bottom-up.

Moving on to methods specifically using LC-MS-based proteomics, **Table 2** summarizes selected studies that were primarily focused on finding or understanding the root causes of the TMZ resistance development. This table was compiled from the Google Scholar Search engine using the following terms: “Proteomics of temozolomide resistance glioma,” “Proteomics of TMZ resistant glioma,” and “Proteomics of temozolomide resistance in GBM.” Most experiments in these studies are focused on comparing tumors from specific cell lines or from patients. Many different research groups have utilized subcellular proteomics as an avenue to find the functional role of proteins from specific subcellular components such as EVs, nucleus, plasma membrane, cytoplasm apart from just GCSs, whole-cell lysate, and tumors. **Table 1** also provides a list of experiments undertaken to test the activity and viability or various cell types. The “results” column summarizes the outcomes of selected experiments in terms of protein action to undermine the TMZ resistance phenotype in different types of samples types including cells, tissues, and tumors. Each experiment outlined in this table is unique in terms of supporting a hypothesis with different pieces of evidence. The table also highlights that instead of only comparing tumors of different subjects or cell lines, studying the intratumoral proteome of GMB is a necessary and complementary approach for understanding the protein diversity at different tumor stages with specific time span (184).

**Table 2:** List of selected studies conducted (from year 2000 to date) to understand the TMZ resistance in human GBM and other types of cancers (TMZ treatment) using LC-MS-based proteomics approach.

Year period published (2000-to date)	Title of study	Types of specimens	Cellular components focused for study	Specific experiments/test and/or instrumentations for LC-MS	Methodology or treatments used for LC-MS raw data analysis	Summary of results	Conclusion
2018	Temozolomide affects Extracellular Vesicles Released by Glioblastoma Cells (143)	Human glioma patient derived GSCs	EVs were enriched by ultracentrifugation		LC-MS/MS, MASCOT, signaling pathways, and PPI (STRING)	319 proteins were identified; 209 proteins in the EV fraction from DMSO-treated cells and 253 in the TMZ-treated group, of which 143 were shared between the two groups. Exclusively 110 proteins from EVs of TMZ-treated GSCs and 66 in the control group.	EVs can accumulate in GBM patient’s biopsies, TMZ modulated GSC released EVs and cell adhesion pathway been enriched.
2018	Identification of Key Candidate Proteins and Pathways Associated with Temozolomide Resistance in Glioblastoma Based on Subcellular Proteomics and Bioinformatical Analysis (144)	Human GBM cell line U87 (ATCC, USA)	Cytoplasmic		MaxQuant (identification and LFQ),STRING (for PPI)	161 differentially expressed proteins (65 upregulated and 96 downregulated)	9 proteins (DHX9, HNRNPR, RPL3, HNRNPA3, SF1, DDX5, EIF5B, BTF3, and RPL8) were associated with TMZ resistance and involved in ribosome and spliceosome signaling pathway.
2012	Protein alterations associated with temozolomide resistance in subclones of human glioblastoma cell lines (174)	Human gliomas, D54-MG, U87-MG, Resistant clones were treated with TMZ for 12 months	Cell lysate	1.Chemo-sensitivity assay (survival of cells) 2.Tunnel assay (to determine apoptosis *via* DNA fragments) 3.Proliferation assay 4.Wound healing and adhesion assay 5.Cell-cycle analysis 6.2-dimensional gel electrophoresis (2-DE) 7.MALDI-TOF/TOF	MASCOT	1.Enhanced cell adhesion and migration in TMZ resistance compared with CTR (control) 2.TMZ resistance is mediated *via* loss of cell-cycle check points and alterations in programmed cell death 3.Seven proteins were found to be associated with TMZ resistance	Upregulation of VIM, CTSD, and P4HB
2019	Quantitative Proteomics Analysis Reveals Nuclear Perturbation in Human Glioma U87 Cells treated with Temozolomide (175)	Human GBM cell line U87, MGMT deficient	Nuclear protein extraction from cell lysate	1.Multienzyme digestion filter aided sample preparation (MED FASP) 2.LC-ESI-MS/MS	MaxQuant (identification and LFQ), OmicsBean, (http://www.omicsbean.cn), Kyoto Encyclopedia of Genes and Genomes (KEGG) pathway (http://www.genome.jp/kegg), and STRING (https://www.stringdb.org)	327 upregulated and 128 downregulated differentially abundant proteins Upregulated proteins were enriched in NER, protein processing in ER, NHEJ, MMR, ubiquitin-mediated proteolysis, and HR Downregulated proteins were enriched in ribosome	Dysregulation in nucleus proteins Protein from ribosomes were downregulated Proteins involved in DNA damage pathways were upregulated Potential for tumor screening purposes.
2015	The metalloprotease-disintegrin ADAM8 contributes to temozolomide chemoresistance and enhanced invasiveness of human glioblastoma cells (176)	U87 and U251 human GBM cell lines	Cell lysate	1.Zymography assay (protein expression) 2.Protein activity assay 3.Invasion assay 4.Quantitative proteomics of cell condition medium 5.ELISA assay 6.Isotopic formaldehyde labeling LC-MS/MS	SEQUEST (for identification) and quantitation using ASAPRatio program	1.MMP-1, -9, -14, and ADAM8 are related with TMZ resistance and invasiveness 2.Inhibition of ADAM8 causes TMZ sensitization in GBM 3.ADAM8 causes TMZ resistance *via* pERK1/2 and/or pAkt signaling	ADAM8 is associated with TMZ resistance and invasion in GBM.
2016	Expression of dynein, cytoplasmic 2, heavy chain 1 (DHC2) associated with glioblastoma cell resistance to temozolomide (177)	U87 and U251 cells human GBM cell lines	Cell lysate	1.Flow cytometric cell-cycle assay 2.2D gel 3.MALDI-TOF/TOF analysis 4.siRNA transfection (to knock down the protein of interests) 5.Study of cytochalasin D (known as cytochalasin) 6.qRT-PCR and immunofluorescence (for validation of protein expression level) 7.Protein extraction, BCA protein quantitation and Western blot analysis 8.Immunohistochemistry 9.Nude mouse tumor xenograft assay	PDQuest software analysis, MASCOT, gene ontology (molecular functions (MF), and cellular component (CC),	Rearrangement of cytoskeleton structure and upregulation of DHC2 observed as a part of TMZ resistant mechanism	1.Reorganization of cytoskeleton structure in response to TMZ resistance 2.DHC2 found as probable molecular target along with TMZ chemotherapy.
2013	Temozolomide-modulated glioma proteome: Role of interleukin-1 receptor-associated kinase-4 (IRAK4) in chemosensitivity (178)	U251 (primary for TMZ sensitivity), U138, and LN229 human GBM cell	Whole-cell lysate used for proteomic	1.Cell line transfection with IRAK4 siRNAs and cell viability assay 2.MTT assay (for cell viability and proliferation) 3.2D DIGE 4.Biological variation analysis 5.In-gel tryptic digestion 6.MALDI-TOF MS	MASCOT (identification)	2D-DIGE: 95 proteins were modulated by TMZ 59 protein spots were upregulated and 36 protein spots were downregulated. MALDI-TOF: DARS, GSS, IRAK4 and BCAS1 upregulated and OPTN was downregulated. IRAK4 inhibits TLR signaling and plays a role in TMZ resistance.	IRAK4 plays a role in determining TMZ sensitization.
2017	Combination of a STAT3 Inhibitor and an mTOR Inhibitor Against a Temozolomide-resistant Glioblastoma Cell Line | Cancer Genomics & Proteomics (179)	U87 human GBM cell line	Whole-cell lysate	1.TMZ resistance development with TMZ-dose-escalation approach up to 150 µM and maintained at 100 µM 2.Cell proliferation WST-1 assay 3.Western blotting 4.Inhibition of YKL-40 gene expression using shRNA in TMZ-resistant cell 5.Inoculation of TMZ resistant cell in male nude mice 6.Whole-exome sequencing	Not applicable	1.Phospho-EGFR, phospho-PIK3, phospho-Akt, phospho-mTOR (S6 and 4E-BP1), and phospho-STAT3 were upregulated in TMZ resistant U87 cells 2.Phospho-raf, phospho-ERK, and phospho-MAPK were downregulated 3.Based on cell proliferation assay, combination therapy of STX-0119, and rapamycin suppresses the proliferation.	Combination therapy of an mTOR inhibitor and STAT3 inhibitor has potential as therapeutics against relapsed TMZ-resistant GBM
2022	Recycling of SLC38A1 to the plasma membrane by DSCR3 promotes acquired temozolomide resistance in glioblastoma (180)	U87 human GBM cell line	Plasma membrane	1. Membrane-cytosolic separation 2. Immunofluorescence 3. Intracranial xenograft tumor modeling	MaxQuant (identification and LFQ), OmicsBean, (http://www.omicsbean.cn), Kyoto Encyclopedia of Genes and Genomes (KEGG) pathway (http://www.genome.jp/kegg) and STRING	1.DSCR3 (Down syndrome critical region protein 3) protein was upregulated with TMZ exposure 2.High level of DSCR3 is associated with reduced survival time 3.Silencing DSCR3 by shDSCR3 shows increased sensitivity for MGMT deficient U87 cells	Recycling of membrane proteins by DSCR3 play vital role TMZ resistance acquisition in GBM cells
2014	Proteomics analysis of melanoma metastases: association between S100A13 expression and chemotherapy resistance (181)	Lymph-node biopsies with metastatic cutaneous melanoma samples patients	Tumor samples	1. Tumor sample collection 2. Protein extraction 3. Protein digestion using trypsin, iTRAQ labeling, strong cation exchange clean-up and narrow-range IEF (isoelectric focusing) 4. LS-MS 5. Immunoblotting (for validation of biomarkers) 6. Immunohistochemistry 7. Statistical analysis	MASCOT version 2.2	1. LC-MS: 14 samples were analyzed including five that responded to chemotherapy and 9 that did not. 3,029 proteins were detected in all samples. Selected 94 proteins were defined by ingenuity pathway analysis including Rho and Rac signaling 2. Immunoblotting: 4 proteins (S100A13, F13A1, CSTB, and ISYNA1) showed the similar proteomics and immunoblotting analyses 3. Immunohistochemistry: Significant S100A13 staining was observed in non-responders versus responders.	S100A13 protein was present in significantly higher level in pretreatment tumor DTIC/TMZ non-responder versus responder group.
2015	The proteomic landscape of glioma stem-like cells (182)	GSCc from human GBM	Whole cell lysate	1. Isolation of GSCs 2. Protein extraction from GSCs 3. nLC-MS/MS	1.PEAKS and MASCOT version 2.3.02 2. DAVID webtool for biological function and gene ontology analysis	1.35 GSC lines were analyzed, and two proteins galectin-1 and EGFR were overexpressed in response to TMZ resistance 2. Level of SYMPK was found to be increased. SYMPK is associated with tumorigenesis and knock out of SYMPK inhibits tumor growth. 3. SRSF2 is associated with chemoresistance	GSC proteins associated with GBM were found including SYMPK, SYVN1, and IL5.
2022	SRPX Emerges as a Potential Tumor Marker in the Extracellular Vesicles of Glioblastoma (183)	1. Human GBM cell lines: IN IN-GB-11, IN-GB-28, IN-GB-29, and IN-GB-9 prepared from fresh tumor biopsies Human GBM U87-MG, 2. U251-MG and HEK293T purchased from ATCC 3. Human primary astrocytes (HPAs) purchased from Applied Biological Materials Inc.	EVs	1. Cell line establishment 2. EVs isolation 3. Nanoparticle tracking analysis (NTA) to determine the average size of particles 4. nLC-MS/MS 5. Immunohistochemistry 6. RNA isolation and RT-qPCR 7. Generation of TMZ resistance cell line 8. Transfection with SRPX siRNAs 9. Cell viability, clonogenic survival and TMZ sensitivity assay 10. Statistical analysis	MASCOT 2.4.1 and Scaffold 5.0.1	1.Only one protein SRPX was detected in GBM derived EVs, which was absent in HPA derived EVs 2. Highest level of SRPX expression was observed for TMZ resistant GBM 3. Endogenous transcript level of ARPX was assessed by RT-qPCR and the U251-MG-R (TMZ-resistant cells) showed higher level of SRPX expression compared with U251-MG-P (parental cells) 4. TMZ treatment for 72 h resulted in an increased level of SRPX mRNA in both cell lines U251-MG-R and U251-MG-P 5. Cell viability tests in various experiments showed that depletion of SRPX inhibits tumor growth 6. Silencing SRPX sensitizes GBM cells to TMZ treatment	SRPX is highly enriched in EVs and plays a role in GBM tumorigenesis

Even with significant advances in cancer diagnosis and treatment options, oncologists still fail to provide maximum clinical benefits to patients due to the development of resistance against various therapies. Tumor cells are capable of choosing alternate mechanisms to sustain resistance against therapies. Consequently, generalized and standard clinical approaches are not useful for better prognosis and enhanced survival. No single chemotherapeutic or concomitant medication can work for all types of GBM tumors. To treat GBM in a timely and effective manner, it becomes essential to inhibit and reverse the resistance mechanism.

Considering this, more vast and in-depth proteomics studies would be beneficial to identify all possible pathways triggered by TMZ treatment to kick off resistance. Initiating a global consortium to work jointly on this topic will be a necessary step to create an expansive proteomics database primarily focused on therapy-resistant cells/tumors. The key advantage provided by proteomics data is to give insight into the molecular features and signature biomarker proteins or metabolites active in relevant pathways. This knowledge is essential for the design of future therapies or to restore the efficacy of currently proven drugs such as TMZ in GBM.

## 7 Concept of Global Proteomics Consortium on Cancer Therapy Resistance

It is indispensable to identify as much as possible the mechanisms that activate resistance against cytotoxic drugs. The functional roles of protein molecules are key in understanding these mechanisms. The Clinical Proteomic Technology Assessment for Cancer (CPTAC) organization has been performing a wide array of proteomics studies, also focused on transcriptomics and genomics, for specific tumor samples (185). However, more global collaborations are needed to support this noble action of creating a global library. Proteomics of therapeutic resistance in cancer has the potential to identify relevant drug-resistant biomarkers with the highest specificity.

In the last section of this review article, it is proposed to create a Global Proteomics Consortium on Cancer Therapy Resistance (GPCCTR). GPCCTR would involve collaborative research to link specific drug-resistant cancer proteomics LC-MS data with multi-dimensional studies on genomics, proteomics, and histology, in correlation with clinical conditions of patients with a timeline.

Some important factors to consider and match between studies would be the cancer phenotype and variations in the administered treatment. Other necessary information would include the time of tumor diagnosis, the initial treatment regime, and the length of various treatments.

**Figure 10:**
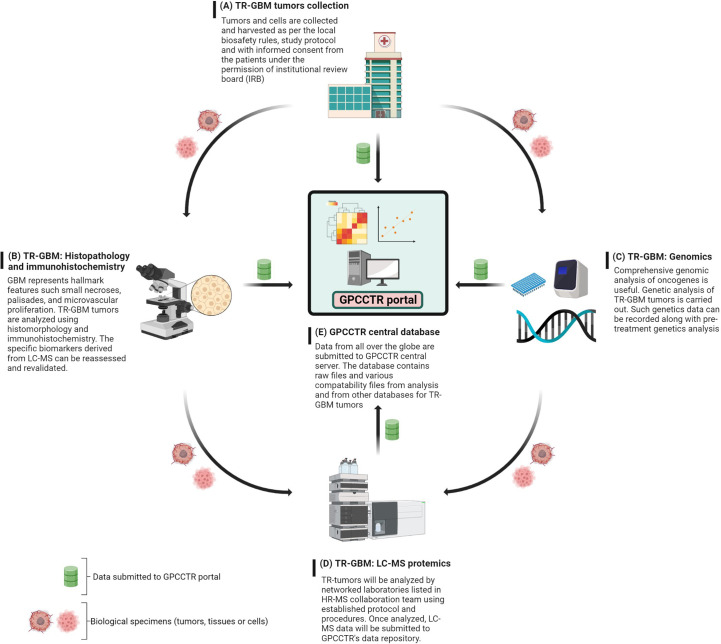
GPCCTR involves five important steps: **(A)** TR-GBM tumor collection: TR tumors from patients during and after treatment will be collected, labeled, and stored as per the scientific protocol. **(B)** TR-GBM histology: TR tumors will be tested for resistant specific markers. **(C)** Genomic analysis of TR-GBM tumors: oncogenic alterations related to TMZ resistance will be analyzed. **(D)** LC-MS proteomic analysis of TR-GBM tumors: TR tumors will be sent to an LC-MS proteomics laboratory where the analysis is performed with established standard methodology. **(E)** GPCCTR central database: all of data from **(A–D)** will be submitted onto the portal. Data analysis and correlation will be performed on a cloud platform. All info and data would be open source and downloadable by registered users.

The foundational work for establishing this consortium could be initiated with a focus on one cancer type such as GBM, and gradual expansion could take place. The main role of GPCCTR would be to consider proteomics data sets for therapy-resistant (TR) tumors with the following main objectives: 1) collection and classification of the TR-GBM tumors based on clinical information; 2) genomic profiling of TR-GBM tumors; and 3) proteomic profiling of TR-GBM tumors that were resistant against radiation, specific chemotherapies, and other molecular therapies. The proposed workflow for GPCCTR is shown in **Figure 10**.

The text below explains steps A through E of the GPCCTR workflow.

A. For the collection of TR-GBM tumors, as per the 2016 WHO classification, these should be classified and named as relevant clinically. It is also critical to mention the duration of treatment patients have undergone. Accurate records of clinical history are beneficial in assessing correlations with proteomics analyses later, where statistics must validate the correlations.
B. Histopathology and immunohistochemistry of TR-GBM tumors: These tumors feature heterogenous histopathology, as well as aggressive proliferation and invasion. Histomorphological patterns are useful in determining the types of secondary structures of GBM cells. Correlations between histopathology and molecular mechanisms can be important indicators on how therapeutic resistance could be overcome (186). The discovery of novel immunohistochemistry-based biomarkers for TR-GBM will then be useful for prediction of the prognosis and optimizing the treatments (187). Data along with the summary of results and a report should be stored in the GPCCTR portal.
C. For the genomic analysis of TR-GBM tumors, information on genomic alteration determines the severity of subgroups of tumors and oncogenic drivers in the development of resistance. Resistance-specific genomic alterations are key to determining the downstream effects on cellular physiology and on the proteome. Genomic analysis after the course of treatment docks additional values for revalidation of LC-MS data and determination of significant changes in genomic-based biomarkers (188). Advances in molecular genetics have equipped clinicians with accurate and detailed understanding of genetic and epigenetic alterations (189).
D. For LC-MS proteomic analysis of TR-GBM, tumors categorized as per sections A and B should be analyzed by LC-MS according to predefined protocols with standard operating procedures (SOPs) in terms of sample preparation and labeling of peptides or amino acids. For labeling, isobaric tags can be used for relative and absolute quantitation: isobaric tags for relative and absolute quantitation (iTRAQ), stable isotope labeling by amino acids in cell culture (SILAC), and TMT (149, 150).
E. The central repository of GPCCTR is used to upload all data collected by the consortium scientists. Data should contain details on clinical history including the treatments or therapies undertaken by the patients, reports on genetic profiling of tumors, and raw/processed data from LC-MS proteomic analyses. The quality of data is critical, and hence, all collaborators are required to follow SOPs to determine the criteria for data to be uploaded into the portal.

## 8 Conclusion

Temozolomide has remained a first-line chemotherapy treatment for GBM for almost two decades. However, intrinsic or acquired resistance development against TMZ diminishes the therapeutic benefits of treatment, resulting in poor prognosis. Various combinations of treatments are utilized with continuous development of new therapies, but the overall prognosis remains poor. Extensive research studies have been conducted to try and elucidate the mechanism(s) of TMZ resistance development and for the development of alternative therapies. Studies show that TMZ resistance development can be driven by multiple mechanisms involving various genes, proteins, and enzymes.

The use of advanced LC-MS-based proteomics to help understand TMZ resistance holds significant promises, as these methods can delve into the molecular understanding of resistance mechanisms, which is necessary to developing personalized treatment and new therapeutics. However, the invasive and heterogenous nature of GBM tumors adds other challenges for neurooncologists in the development of universal treatment plans. A global effort is needed to conduct a multidimensional proteomic study by laboratories around the world, in order to acquire a profound understanding of TMZ resistance. The proposed GPCCTR global database would include all clinical variables. Vast proteomics datasets can provide a deep understanding of specific proteins and pathways involved in TMZ resistance. Studying an extensive number of TMZ-resistant tumors using LC-MS-based proteomics can reveal the potential mechanisms or pathways by which the resistance phenotype is developed. The GPCCTR database is a unique concept to link global experts in proteomics and oncology and put together their contributions into an expansive data library. This would be highly beneficial for the identification of new targets and for the design of new drugs, including personalized medicine.

## Author contributions

MT: concept, extensive search for literatures, creation of figures (from **Figures 1**–**10**) and drafted this manuscript; VC: critical review of the manuscript, inputs and editing; HP: critical review of the manuscript, inputs and editing as subject expert including correctness and grammar and final approval. All authors contributed to the article and approved the submitted version. 
